# Hydrogen alleviated cognitive impairment and blood‒brain barrier damage in sepsis-associated encephalopathy by regulating ABC efflux transporters in a PPARα-dependent manner

**DOI:** 10.1186/s12868-023-00795-3

**Published:** 2023-07-20

**Authors:** Yuanyuan Bai, Wen Mi, Xiaoyin Meng, Beibei Dong, Yi Jiang, Yuechun Lu, Yonghao Yu

**Affiliations:** 1 Department of Anesthesiology, Tianjin Institute of Anesthesiology, Tianjin Medical University General Hospital, No. 154 Anshan Road, Heping District, Tianjin, 300052 PR China; 2 Department of Anesthesiology, Tianjin Baodi Hospital, Baodi Clinical College of Tianjin Medical University, Tianjin, 301800 China; 3 Department of Gynecology and Obstetrics, Tianjin Medical University General Hospital, Tianjin, 300052 China; 4 Department of Anesthesiology, The Second Hospital of Tianjin Medical University, Tianjin, 300211 China

**Keywords:** Blood‒brain barrier, Peroxisome proliferator-activated receptors, ABC efflux transporters, Sepsis-associated encephalopathy

## Abstract

Hydrogen (H_2_) can protect against blood‒brain barrier (BBB) damage in sepsis-associated encephalopathy (SAE), but the mechanism is still unclear. We examined whether it is related to PPARα and its regulatory targets, ABC efflux transporters. After injection with DMSO/GW6471 (a PPARα inhibitor), the mice subjected to sham/caecal ligation and puncture (CLP) surgery were treated with H_2_ for 60 min postoperation. Additionally, bEnd.3 cells were grown in DMSO/GW6471-containing or saline medium with LPS. In addition to the survival rates, cognitive function was assessed using the Y-maze and fear conditioning tests. Brain tissues were stained with TUNEL and Nissl staining. Additionally, inflammatory mediators (TNF-α, IL-6, HMGB1, and IL-1β) were evaluated with ELISA, and PPARα, ZO-1, occludin, VE-cadherin, P-gp, BCRP and MRP2 were detected using Western blotting. BBB destruction was assessed by brain water content and Evans blue (EB) extravasation. Finally, we found that H_2_ improved survival rates and brain dysfunction and decreased inflammatory cytokines. Furthermore, H_2_ decreased water content in the brain and EB extravasation and increased ZO-1, occludin, VE-cadherin and ABC efflux transporters regulated by PPARα. Thus, we concluded that H_2_ decreases BBB permeability to protect against brain dysfunction in sepsis; this effect is mediated by PPARα and its regulation of ABC efflux transporters.

**Supplementary Information:**

The online version contains supplementary material available at 10.1186/s12868-023-00795-3.

## Introduction

Known as a syndrome of systemic inflammation, sepsis resulting from various infectious factors is a main public health problem and the major cause of death in critically ill patients [1, 2]. Many patients with sepsis develop a form of cognitive impairment named sepsis-associated encephalopathy (SAE) [3], which can lead to acute or long-term mortality [4]. However, the pathophysiology of SAE is too complex to clarify, and studies have indicated that it is associated with brain injury, especially blood‒brain barrier (BBB) impairment [3–5].

A series of microvascular endothelial cells constitute the BBB, which regulates the molecular permeation between the peripheral circulatory system and the central nervous system (CNS) [6]. Intercellular tight junction proteins and ABC efflux transporters (ATP-binding cassette efflux transporters) play a key role in maintaining the function and integrity of the BBB [7–10]. BBB dysfunction involving ABC efflux transporters has been demonstrated to be a crucial early event in CNS disease pathogenesis [11–13]. It results greatly in SAE because the CNS cannot defend against neurotoxic substances, including inflammatory mediators, free radicals, circulating leukocytes and so on [14, 15]. Furthermore, barrier damage leads to brain oedema and perfusion reduction of the microvasculature, resulting in and aggravating the loss of neurons in SAE [15]. Thus, BBB integrity protection is deemed a vital treatment for all kinds of diseases in the CNS [16].

Regarded as a selective antioxidant, hydrogen (H_2_) can treat over 70 diseases [17]. We have already suggested that hydrogen inhalation or hydrogen-rich water intake is an effective treatment for organ damage in sepsis (including intestine, lung, liver and brain damage) [18–21]. We also illustrated that 2% H_2_ could relieve cognitive impairment owing to sepsis in rodent models [21, 22]. Nevertheless, the H_2−_specific mechanism is still unclear. It was proven that the inhalation of H_2_ showed a significant protective effect on BBB dysfunction and corresponding brain injury in sepsis [21]. Thus, protection of the BBB may be a vital target for the treatment of SAE with hydrogen.

Known as a type II nuclear receptor, peroxisome proliferator-activated receptor (PPARα) is related to glucose homeostasis, lipid metabolism, the inflammatory response, cell differentiation, the cellular response and apoptosis [23]. Additionally, PPARα plays a key role in the regulation of the NF-κB signalling pathway, decreasing the level of inflammatory mediators and the oxidative stress response, promoting neurogenesis and cell differentiation in the CNS, and showing a protective effect in neuroinflammatory lesions, including neurodegeneration [24]. In human hepatoma cells, PPARα also regulates hepatic genes, including multidrug resistance protein 1 (MDR1), multidrug resistance-associated protein 2 (MRP2/ABCC2), MRP3, and MRP4, which encode ABC transporters known to play a vital role in effluxing bile acids and therapeutic drugs [25–27]. A recent study with cultured human brain capillary endothelial cells showed that exposure to clofibrate (one of the PPARα ligands) increased the expression and transport activity of breast cancer resistance protein (Bcrp/ABCG2), demonstrating that drug efflux transporters at the BBB may be PPARα targets [28]. Importantly, the present study has shown that the activation of PPARα regulates the expression of ABC efflux transporters P-gp/ABCB1, Bcrp and Mrp2 in the BBB to affect the activity of exogenous and endogenous small molecule substances in brain tissues, regulates blood‒brain barrier permeability and participates in the mechanical regulation of information communication between the brain and other organs [9, 29]. We have shown that PPARα mediates the CREB-BDNF signalling pathway to alleviate cognitive dysfunction in septic mice treated with H_2_ [30]. Thus, based on previous research, our study explored the potential protective role of H_2_ in brain damage in sepsis and whether the effects were due to the upregulation of PPARα to ABC efflux transporters.

## Materials and methods

### Experimental procedure

**Fig. 1:**
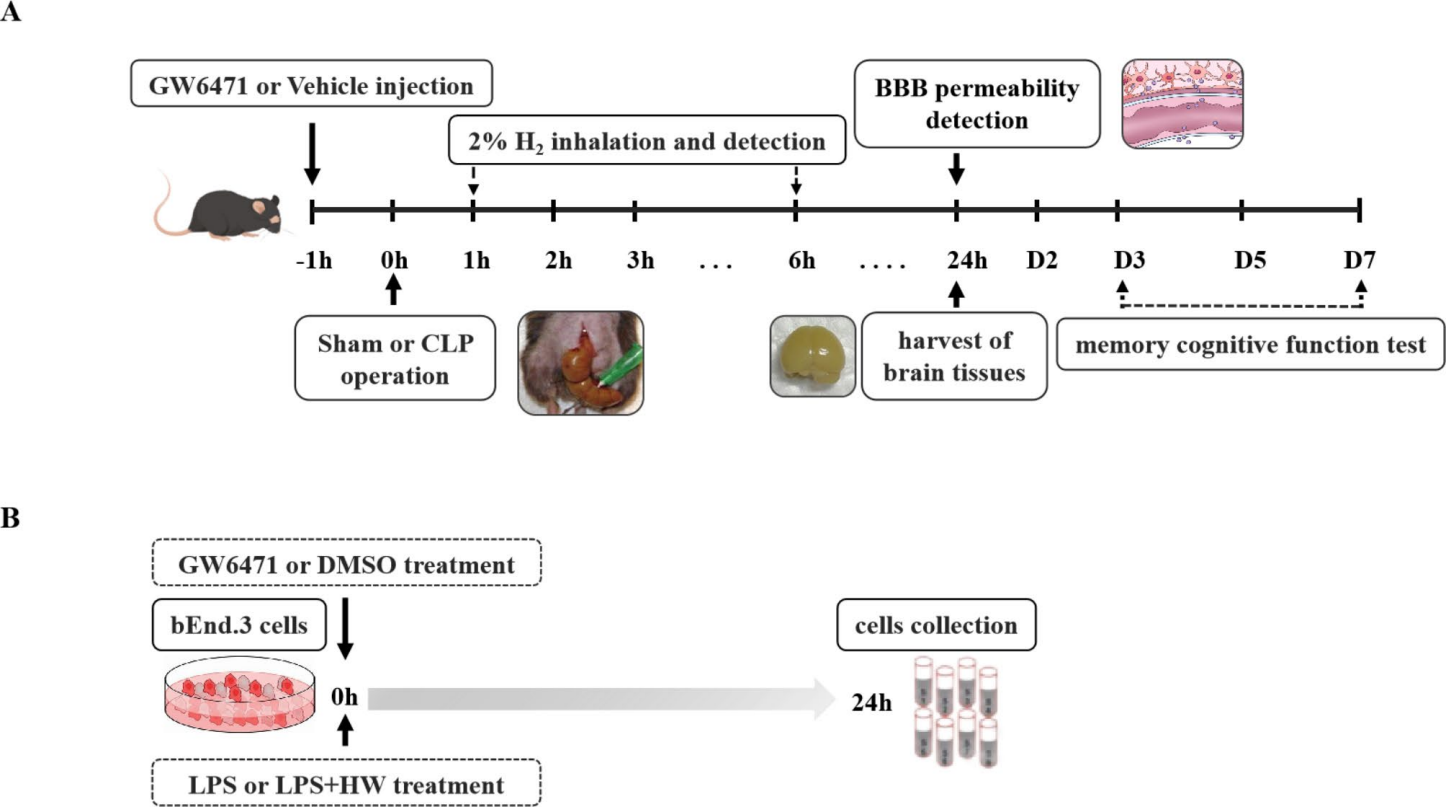
Experimental design. **(A)** C57BL/6J mice (6–8 w, 20–25 g) were subjected to sham or CLP operation. One hour after the injection of GW6471 or vehicle, sham and CLP operations were conducted. H2 or fresh air was inhaled for 60 min starting from 1 and 6 h postoperation with H2 concentration detection. The brain tissue of different groups was obtained for tests 24 h after the sham or CLP procedure. Memory cognitive function tests were conducted from 24 h to 7 days after the sham or CLP procedure. Different groups of brain tissues were used for all the examinations as described in the Materials and Methods. **(B)** Mouse bEnd.3 cells were incubated with control + DMSO or GW6471 medium, control + HW + DMSO or GW6471 medium, LPS + DMSO or GW6471 medium, and LPS + HW + DMSO or GW6471 medium. The cells and culture medium supernatant were collected for testing 24 h after incubation. CLP, caecal ligation and puncture; LPS, lipopolysaccharide; GW6471, a PPARα inhibitor

In vivo study (Fig. 1A): C57BL/6J male mice (6–8 w, 20–25 g) were divided randomly into four groups: Sham, CLP, CLP + H_2_ and CLP + H_2_ + GW6471 groups. Sham or CLP surgeries were performed, and the H_2_ and GW6471 groups were treated with H_2_ gas (for 60 min at 1 and 6 h postsurgery) and 20 mg/kg GW6471 (intraperitoneal injection at 1 h preoperation), respectively. Following the operations, the survival rates of all groups were recorded, and some mice were euthanized 24 h postoperation with isoflurane, and brain tissue (cortex) was harvested. In addition, each group of mice was subjected to the fear conditioning test (n = 5) and Y-maze test (n = 3) (1–7 days after the procedure). Cortical tissues in mice were used for Evans blue extravasation and brain water content determination. The cortex tissues were used for P-gp/Abcb1, breast cancer resistance protein (Bcrp/Abcb11), multidrug resistance-associated protein 2 (Mrp2/Abcc2), VE-cadherin, occludin and ZO-1 detection with WB. Brain slices from mice (n = 3) were obtained for TUNEL staining and Nissl staining. The cortex tissues per group (n = 6) were subjected to an enzyme-linked immunosorbent assay (ELISA) to evaluate inflammatory mediators (TNF-α, IL-6, IL-1β, and HMGB1).

In vitro study (Fig. 1B): bEnd.3 cells were treated with the following conditions: control + DMSO, LPS + DMSO, control + H_2_ + DMSO, LPS + H_2_ + DMSO, control + GW6471 (PPARα antagonist), LPS + GW6471, control + H_2_ + GW6471, and LPS + H_2_ + GW6471. To clarify the vital effects of PPARα, either 25 µM GW6471 (a PPARα antagonist) dissolved in DMSO (MCE, Tocris Bioscience, USA) or DMSO without GW6471 was added to DMEM without FBS, and the cells in all groups were added to 1 µg/ml LPS (Cat# 4394, Sigma, USA) or the same volume of PBS/saline for 24 h. The LPS and control groups (DMSO or GW6471) were cultured in normal DMEM without FBS, and the control and LPS groups (+ H_2_ + DMSO or GW6471) were developed in hydrogen-rich DMEM without FBS for 24 h. In all groups, the cells were harvested following the corresponding procedure at 24 h and centrifuged at 10,000 × g at 4 °C for 10 min. The liquid supernatant was obtained for Western blot analysis.

### bEnd.3 cell cultivation

Purchased from the American Type Culture Collection (ATCC), the bEnd.3 lines were derived from brain-endothelial cells in mice and were developed in Dulbecco’s modified Eagle’s medium (DMEM, Cat# PM150210, Procell, China) with 10% foetal bovine serum (FBS, Cat# 164210-50, Procell, China) and 1% streptomycin/penicillin solution (Cat# PB180120, Procell, China). Cultured in a 5% CO_2_ humid atmosphere at 37 °C, the second generations were applied to the experiment when the bEnd.3 cells grew to 80% confluence in a Petri dish, and the subculture was administered.

### Ethics statement and animal preparation

Our research protocols were carried out with the permission of the Animal Experimental Ethics Committee of Tianjin Medical University General Hospital (No.IRB2020-DW-18) (Tianjin, China). Male C57BL/6J mice in this study were provided by the Laboratory Animal Center of the Military Medical Science Academy (Beijing, China) and were used in accordance with the National Institutes of Health Guide for Care and Use of Laboratory Animals. The controlled conditions were suitable for the mice (a humidity: 55 − 65%; temperature: 20-25 °C; 12/12-hour light-dark cycle) and given ad libitum water and food.

### Caecal ligation and puncture (CLP)

In our previous study, we chose the CLP model for the establishment of sepsis in mice [30]. After adaptation for 7 days in the laboratory environment, the mice were anaesthetized with isoflurane and placed in the supine position. After sterilizing the skin in the abdomen and making a 1 cm incision, 35% of the caecum was ligated after exposure. Next, a 21-gauge needle was used to puncture twice the caecum, and sterile forceps were applied to push out approximately 0.3 mL of caecal content. Finally, the caecum was placed back into the abdominal cavity, and the skin and muscles were stitched. In contrast, the Sham group merely had the caecum exposed and then had the abdominal cavity closed. After the operation, saline (1 mL) was subcutaneously applied to the mice with lidocaine cream (Ziguang, Beijing, Cat# H20063466) to relieve suffering. After the procedures, the animals were placed in a 20-25 °C room with a heating blanket.

### Intraperitoneal injection of GW6471

GW6471 (Tocris Bioscience) was dissolved in DMSO to a concentration of 12.5 mg/ml. A 20 mg/kg dose of GW6471 was intraperitoneally injected into mice 1 h before the operation [31].

### Hydrogen-rich medium

We made the hydrogen-rich medium (HM) with the method mentioned in our previous studies [32, 33]. Briefly, with 0.4 MPa pressure conditions, the mixture of H_2_ (1 l/min) and air (1 l/min) was dissolved in 5.6 mM glucose DMEM for at least 4 h to obtain supersaturation (0.6 mM H_2_). A TF-1 gas flow metre (Tokyo, Japan, Yutaka Engineering Corp.) was used to produce H_2_. A special sealed aluminium vacuum package bag was used to store the prepared HM at 4 °C under atmospheric conditions. The medium must be freshly prepared every 7 days to keep the H_2_ concentration saturated.

### Hydrogen inhalation

A box with two outlets for H_2_ outflow and inflow was applied for the treatment of H_2_ for 1 h in mice at 1 and 6 h postsurgery. The mixture of H_2_ and air was produced and infused into the box at a 4 l/min rate with a TF-1 gas flow metre (Yutaka Engineering Corp., Tokyo, Japan). A detector (HY-ALERTA Handheld Detector, model 500, H_2_ Scan, Valencia, Calif) was applied to continuously monitor the concentration of H_2_ maintained at 2% throughout treatment in the box. Carbon dioxide was dissolved by baralyme in the box and discharged. The animals treated without H_2_ were placed in the same type of box with room air [19].

### Survival rates

As described previously, we recorded the survival rates of mice 7 days postoperation [18]. The experimental procedures were conducted three times.

### Y-maze test and contextual fear conditioning test

The Y-maze was composed of A, B, and C arms with an angle of 120° from each other, which recorded the number of alternations/times that every mouse strolled into all three arms in a row without visiting one arm twice, i.e., for example, the pattern of ABC, BCA, or CAB. All mice were placed into the maze centre and given free activity in all arms for 10 min. To record the number of alternations and line crossings, we used the ANY-maze video tracking system (Stoelting, USA), and then we analysed the mouse activity to calculate the alternation percentage [30, 34].

Contextual fear conditioning test: This test is widely applied for evaluating memory functions [21, 35, 36]. The fear conditioning test contained three stages: the part of habituation, the part of training, and the part of the test. In the habituation phase, mice were put into the training context with free movement for 10 min. In the training phase, each group of mice was placed in a fear chamber one day before modelling, acclimated for 2 min, and then given 20 s of a single-frequency sound signal (70 dB) coterminating with a foot shock (0.70 mA, 2 s). After an interval of 25 s, the auditory stimulus was played for another 60 s coterminating with a second foot shock, marking the end of one full cycle of training (105 s). Six cycles of training were administered. The mice showed panic, escape, or rigidity when they heard the sound signal (no other motor behaviour except breathing) and squeal, jump and escape when they were shocked, indicating the formation of fear memory. The sham or CLP model was established the day after fear memory formation. Contextual fear memory tests were administered at 1 d, 2 d, 3 d, 5 d and 7 d after sham surgery or CLP. During the test period, the mice in each group were put into the fear box, which was the same as the environment during the training period, but were not given the sound signal and electrical stimulation and were given free movement for 300 s. The analysis system of ANY-maze video was applied to the time of rigidity record in each group during the training period and the test period, and the percentage of time of rigidity was calculated. The mice were regarded as freezing if there was no movement for 2 s (freezing time/300 s×100%= freezing time ratio).

### Detection of inflammatory cytokines

Cortical tissues in all groups were homogenized, centrifuged at 10,000 × g at 4 °C for 10 min and collected with the supernatant. The levels of IL-1β, TNF-α, IL-6 (Cat# RLB00; Cat# RTA00; Cat# R6000B; R&D Systems, Inc.) and HMGB1 (ARG81310; Arigo) were measured by ELISA kits based on the manufacturer’s instructions.

### TUNEL staining

With the TUNEL assay, neuronal apoptosis was observed at 24 h postsurgery. Fluorescein-dNTPs and fluorescein-dUTP compose the nucleotide-labelling mix (TUNEL reagent) and are used for in situ analysis of apoptosis. The preparation of the TUNEL reaction mixture requires the combination of the nucleotide-labelling mix and the TUNEL enzyme. The cells with broken DNA strands were labelled by the reaction mixture, providing for analysing and quantifying the extent of apoptosis at the single-cell level. The cell nuclei were stained with DAPI to become blue, and apoptotic cells were stained green.

### Nissl staining

Twenty-four hours after the operations, the animals’ brains were obtained for the observation of the extent of brain injury. Brain tissues were obtained after transcardial perfusion with 4% paraformaldehyde, postfixed for 24 h with formalin-free fixative and then embedded in paraffin. Following rehydration and deparaffinization with ethanol and dimethyl benzene, 10 μm brain sections were used for Nissl staining. The damage in the cortex region was assessed with a microscope (CKX41, Tokyo, Japan).

### EB extravasation in the blood‒brain barrier

As just described, the BBB permeability was evaluated by the extravasation of EB dye [21]. At 24 h postoperation being anaesthetized, the mice were treated with a 2.5 mL/kg dose of EB (the concentration of 2%, Cat# R31047, Shanghai Yuan Ye Bio-Technology Co., Ltd, China) through the tail vein and observed for 2 h. Then, all the animals were euthanized and transcardially perfused with a large amount of saline. The cortex of every mouse was collected, weighed and incubated for 48 h at 37 °C after homogenization in 1 mL formamide. After centrifugation, the optical density at 620 nm of the supernatant liquid was calculated with a microplate reader. Based on a linear standard curve, a large quantity of EB was quantified (µg/g wet weight) and then shown as the relative amount [32].

### Brain water content (WC)

The animals were euthanized at 24 h to harvest whole brain tissues after surgery. The water content in the brain was assessed with a dry‒wet method as described in previous research [21]. After being weighed instantly, the brain tissues were dried at 100 °C in an oven for 24 h, and then the wet weight and dry weight were obtained. Based on the following formula, WC = 100% × [wet weight/dry weight].

### Western blot (WB)

WB was used to evaluate the expression of PPARα, MRP2, P-gp, BCRP, ZO-1, VE-cadherin and occludin. Twenty-four hours after the operation, the cortex tissue from each group was collected and weighed. The protease inhibitor and PBS were added to each sample, and after centrifugation at 4 °C for 15 min at 15,000 × g, we obtained the supernatants and then quantified the collected protein concentrations. The samples were added to the loading buffer (Beijing Solarbio Science & Technology Co., Ltd. ), boiled and denatured. The proteins were separated by sodium dodecyl sulfate‒polyacrylamide gel electrophoresis (SDS‒PAGE) and electrotransferred onto polyvinylidene fluoride membranes (Millipore, Germany). Next, the membranes were soaked in Tris-buffered saline with Tween (TBST) with 5% nonfat milk for 2 h and incubated in the following primary antibodies at 4 °C overnight: PPARα, MRP2, P-gp, BCRP (1:1000, Cat# ab61182; ab203397; ab170904; ab130244, Abcam, Britain), ZO-1 (1:1000, Cat#AF5145, Affinity, USA), VE-cadherin (1:500, Cat# AF6265, Affinity, USA), and occludin (1:1000, Cat# DF7504, Affinity, USA). After washing with TBST, the membrane was soaked in TBST containing goat anti-rabbit (1:5000, Cat# 31,466, Invitrogen, USA) or anti-mouse antibodies (1:5000, Cat# 31,430, Invitrogen, USA) for 1 h at room temperature. The nitrocellulose membrane was immersed in electrochemiluminescence (ECL) reagent. The protein bands were analysed by ImageJ. The levels of the target proteins were standardized to β-actin or GAPDH.

### Statistical analysis

GraphPad Prism 8.0 and SPSS 21.0 software were used to analyse the data. The Shapiro‒Wilk test and KS normality test were used to determine whether a sample came from a normal distribution. The survival rates were assessed as percentages and then analysed by the log‑rank (Mantel‒Cox) test. Data from the behavioural test and H_2_ concentration detection were analysed using two-way analysis of variance (ANOVA) with Tukey’s multiple comparisons test, and the ELISA, Western blot, Evans blue extravasation and brain water content data were analysed by one-way ANOVA with Tukey’s multiple comparisons test. Quantifiable data are expressed as the mean ± standard deviation (SD). P < 0.05 was recognized as statistically significant in all tests.

## Results

### PPARα decreases the expression of inflammatory mediators in cortex tissues and increases the survival rates of septic mice treated with H_2_

**Fig. 2:**
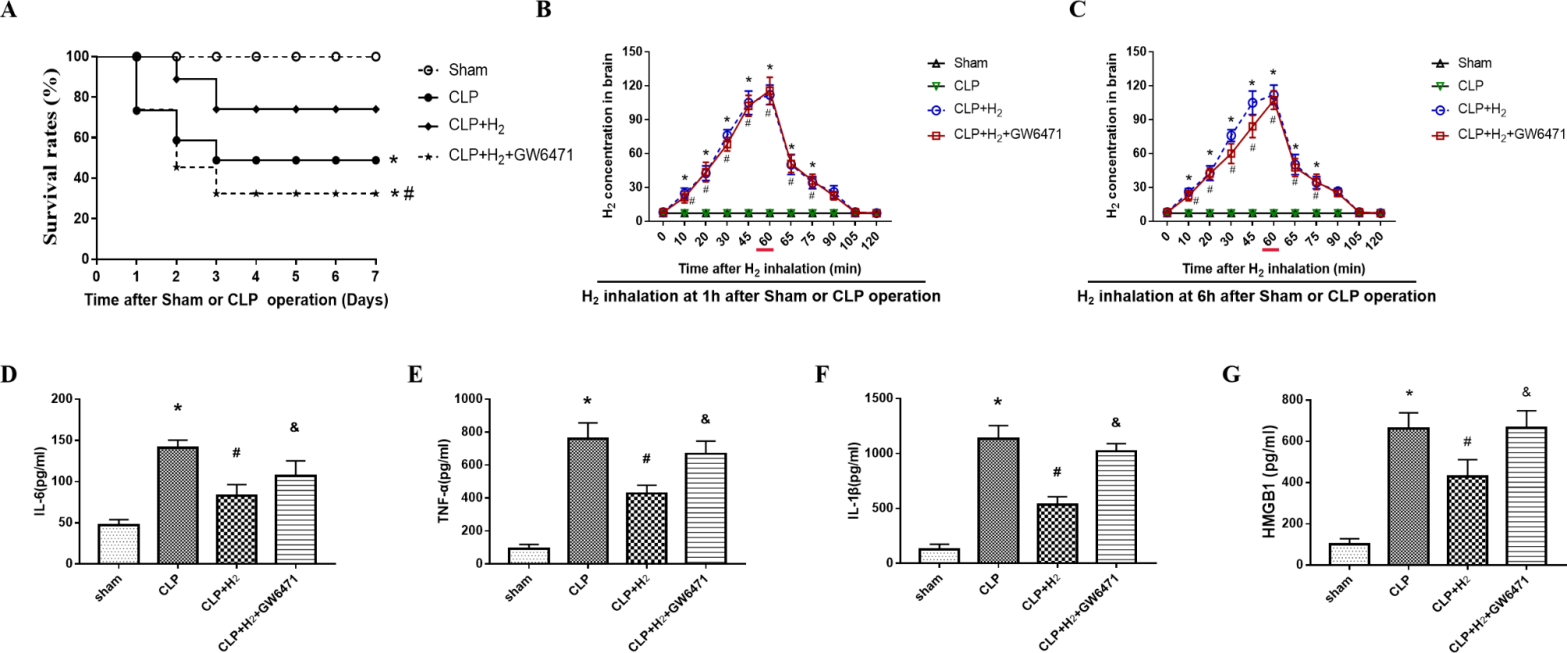
Treatment with 2% hydrogen improved the 7-day survival rate and decreased the levels of inflammatory cytokines in septic mouse cortex tissues. Animals inhaled 2% H_2_ for 60 min starting at 1 and 6 h after the sham or CLP operation, respectively, and the H_2_ concentration was recorded. **(A)** The survival rate was analysed at 1, 2, 3, 5 and 7 days postoperation. Compared with the sham group, the survival rates of the CLP, CLP + H_2_ and CLP + H_2_ + GW6471 groups were significantly decreased on day 7 (100% vs. 50%, 100% vs. 70% and 100% vs. 30%). Values are expressed as survival percentages (n = 20). **(B)** At 0, 10, 20, 30, 45, 60, 65,75, 90, 105, and 120 min after the start of H_2_ inhalation and **(C)** at 5, 15, 30, 45, and 60 min after the termination of H_2_ treatment in all groups of mice. Data are expressed as the mean ± SD (n = 3). **(D-G)** The cortex tissues were harvested to measure inflammatory cytokines (IL-1β, TNF-α, IL-6 and HMGB1) (n = 6). *P < 0.05 vs. sham group; #P < 0.05 vs. CLP group; &P < 0.05 vs. CLP + H2 group

The survival rate in the Sham group was 100% (Fig. 2A), while the survival rate in the caecal ligation and puncture (CLP), CLP + H_2_ and CLP + H_2_ + GW6471 groups decreased (50% vs. 100%, 70% vs. 100%, 30% vs. 100%: P < 0.05). The inhalation of 2% H_2_ was related to PPARα and markedly ameliorated the survival rate in CLP mice (50% vs. 70%, 30% vs. 70%: P < 0.05). In addition, at 1 and 6 h postoperation, the concentration of H_2_ in septic mouse brains was measured. As shown in Fig. 2B C, the concentration of H_2_ in the CLP + H_2_ and CLP + H_2_ + GW6471 groups increased within the first 60 min with H_2_ inhalation (P < 0.05 vs. sham group; P < 0.05 vs. CLP group; all at 10, 20, 30, 45, 60 min) and peaked at almost 45 min, and the maximum concentration was maintained for 15 min until the termination of H_2_ treatment in the mouse brain. When H_2_ inhalation was stopped, its concentration in the CLP + H_2_ and CLP + H_2_ + GW6471 groups decreased rapidly (P < 0.05 vs. CLP group at 65 and 75 min). However, there was no significant difference between the CLP + H_2_ + GW6471 and CLP + H_2_ groups (P > 0.05). Then, we assessed the expression of some important inflammatory cytokines to reveal the anti-inflammatory effect of PPARα in CLP mice treated with H_2_ using ELISA. Interleukin (IL)-1β, tumour necrosis factor (TNFα), high mobility group protein 1 (HMGB1) and IL-6 are the most vital cytokines promoting the inflammatory response in sepsis and are key biomarkers of septic shock in systemic inflammatory response syndrome [37, 38]. In particular, IL-6 plays a crucial role in BBB permeability and tight junction protein levels [39]. The release of inflammatory cytokines (IL-1β, HMGB1, IL-6 and TNF-α) was increased in the CLP, CLP + H_2_ + GW6471 and CLP + H_2_ groups (P < 0.05, Fig. 2D-G) compared to the sham group. However, the increasing levels of IL-1β, HMGB1, IL-6 and TNF-α in the CLP + H_2_ group were obviously less than those in the CLP + H_2_ + GW6471 group (P < 0.05). In addition, the inhalation of H_2_ upregulated the PPARα level and slightly reduced IL-1β, IL-6 TNF-α and HMGB1 expression. Thus, PPARα exerts necessary effects on the inhibition of neuroinflammation during sepsis induced by H_2_.

### PPARα upregulation by H_2_ improves CLP-induced neurobehavioural changes in mice

**Fig. 3:**
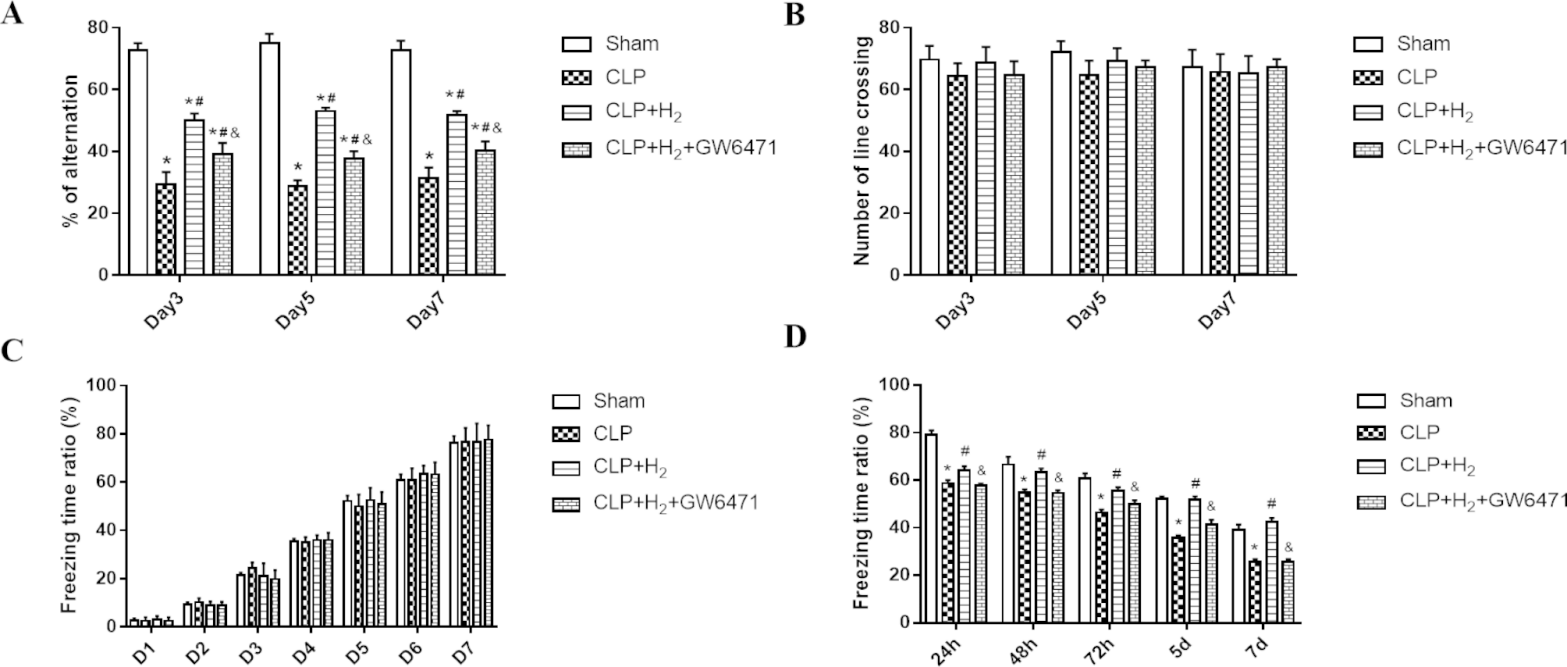
Treatment with 2% H_2_ related to PPARα improved the spontaneous alternation test in the Y-maze and fear conditioning memory of CLP-induced septic mice from days 1 to 7. **(A)** Cognition function 3, 5 and 7 days after the operation was assessed using the Y-maze test (n = 3). The percentage of alternation was determined with the spontaneous alternation test, and **(B)** the number of line crossings was unchanged. **(C)**. The acquisition of memory during the training phase before the operation was similar among all groups in the fear conditioning test (n = 5). **(D).** Fear conditioning memory was quantified by the percentage of freezing time that was improved in the CLP + H_2_ group at days 1 to 7 after CLP operation versus the CLP and CLP + H_2_ + GW6471 groups. CLP, caecal ligation and puncture; SD, standard deviation. Values are expressed as the mean ± SD. *P < 0.05 versus the sham group; #P < 0.05 versus the CLP group; &P < 0.05 versus the CLP + H_2_ group

In the Y-maze spontaneous alternation test, which evaluates the memory and learning ability of the mice (Fig. 3A and B), the alternation percentage was reduced in the CLP and CLP + H_2_ groups compared to the Sham group on postoperative days 3, 5, and 7, whereas the alternation percentage in the CLP + H_2_ group was higher than that in the CLP + H_2_ + GW6471 and CLP groups (Fig. 3A; P < 0.05). In terms of the number of crossings, none of the 4 groups showed statistically significant differences (Fig. 3B; P > 0.05). Additionally, the fear conditioning test was applied to evaluate the environmental correlation of the fear conditions in mice. The fear conditioning test showed that fear memory quantified by the freezing time percentage in the CLP + H_2_ group was improved compared to that in the CLP + H_2_ + GW6471 and CLP groups (P < 0.05, Fig. 3C and D). Analysing the data in the results, hydrogen gas improved the neurobehavioural changes in septic mice, which was closely associated with the upregulation of PPARα.

### Hydrogen improves BBB neuron dysfunction in CLP model mice via PPARα

**Fig. 4:**
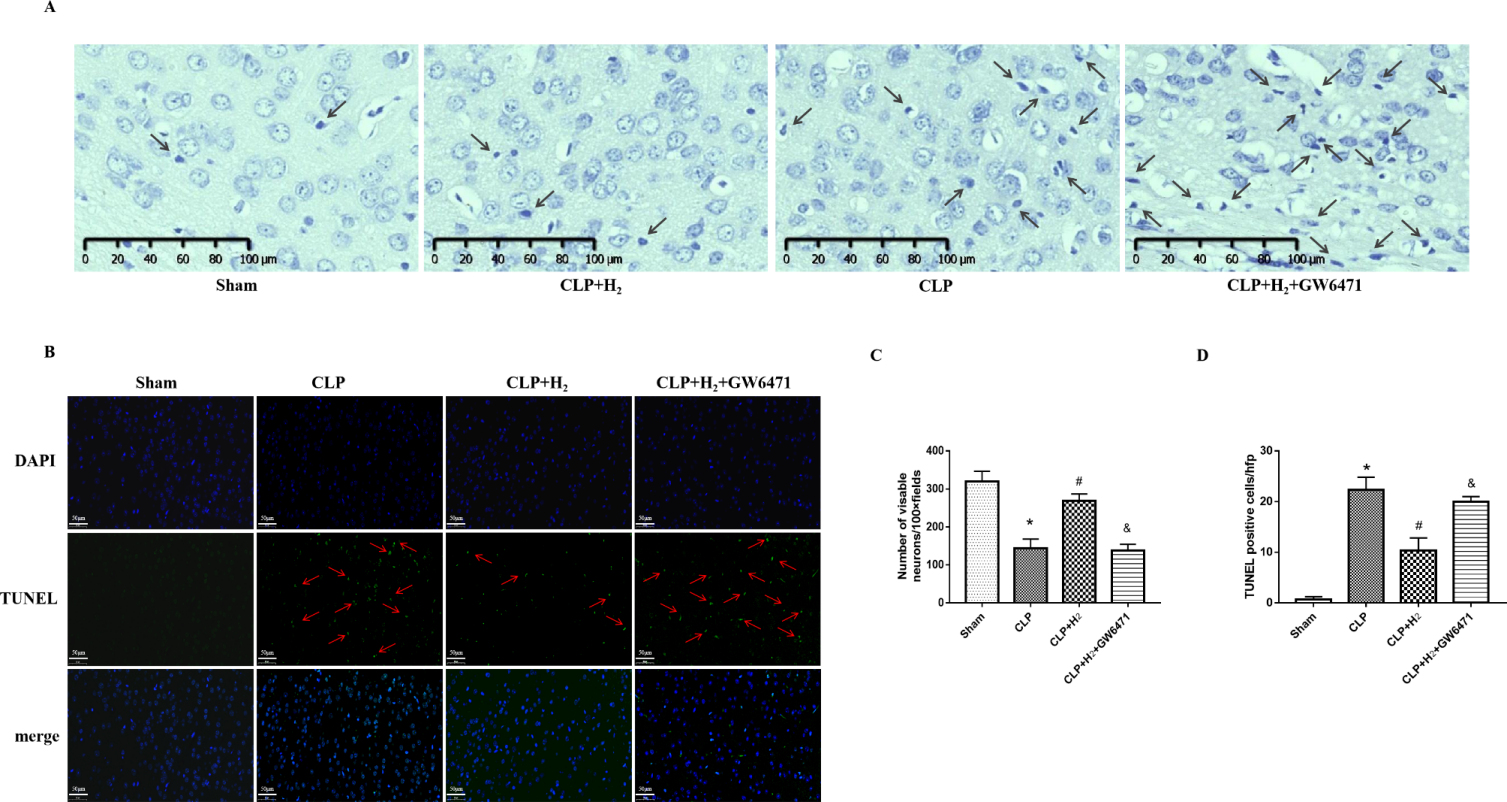
Treatment with 2% H_2_ related to PPARα mitigated the histopathologic changes and prevented neuronal apoptosis in septic mouse brain tissues. **(A)** and **(C)** Regular morphology of the pyramidal neurons in the Sham group was observed compared to the other groups. Many damaged neurons (marked with arrow), in which the Nissl body was decreasing or dissolving, were observed in the CLP and CLP + H_2_ + GW6471 groups. Neurons were significantly improved in the CLP + H_2_ group vs. the CLP and CLP + H_2_ + GW6471 groups. Scale bar = 100 μm. **(B)** Brain samples were harvested to measure TUNEL staining at 24 h after sham or CLP operation, and the TUNEL-positive cells per high-power field were counted **(D)**. Apoptotic cells were stained green in the nuclei (marked with an arrow). DNA in all cells was stained blue with DAPI. Numerous green-stained neurons were observed in the CLP and CLP + H_2_ + GW6471 groups vs. the CLP + H_2_ group, which were significantly decreased by 2% H_2_ inhalation close to PPARα. Scale bar = 50 μm. *P < 0.05 vs. Sham group; #P < 0.05 vs. CLP group; &P < 0.05 vs. CLP + H_2_ group

In this study, TUNEL and Nissl staining were applied to assess the survival of cortical neurons related to PPARα in H_2_-treated septic mice. Nissl staining is shown in Fig. 4A C. The cortex neurons in the Sham group were markedly discernible and had abundant Nissl bodies in their cytoplasm. In the CLP + H_2_ + GW6471 and CLP groups, the pyramidal neurons were sparse and disordered, and their Nissl bodies were noticeably dissolved and fewer in number. In addition, the cortex neurons in the CLP + H_2_ group manifested less disorder than those in the CLP and CLP + H_2_ + GW6471 groups. We further investigated the effects of the inhalation of 2% H_2_ on apoptosis in the cortex region in CLP mice (Fig. 4B and D). In the Sham group, little apoptosis was detected with TUNEL staining. In contrast, there were numerous TUNEL-positive neurons in the CLP + H_2_ + GW6471 and CLP groups. Nevertheless, little neurons were stained with TUNEL in CLP mice with H_2_. The results of Nissl staining and TUNEL staining revealed that PPARα induced by H_2_ exerts an antiapoptotic effect on the cortical neurons of CLP mice.

### Treatment with 2% H_2_ and PPARα attenuated blood‒brain barrier disruption and reduced brain water content in CLP mice

**Fig. 5:**
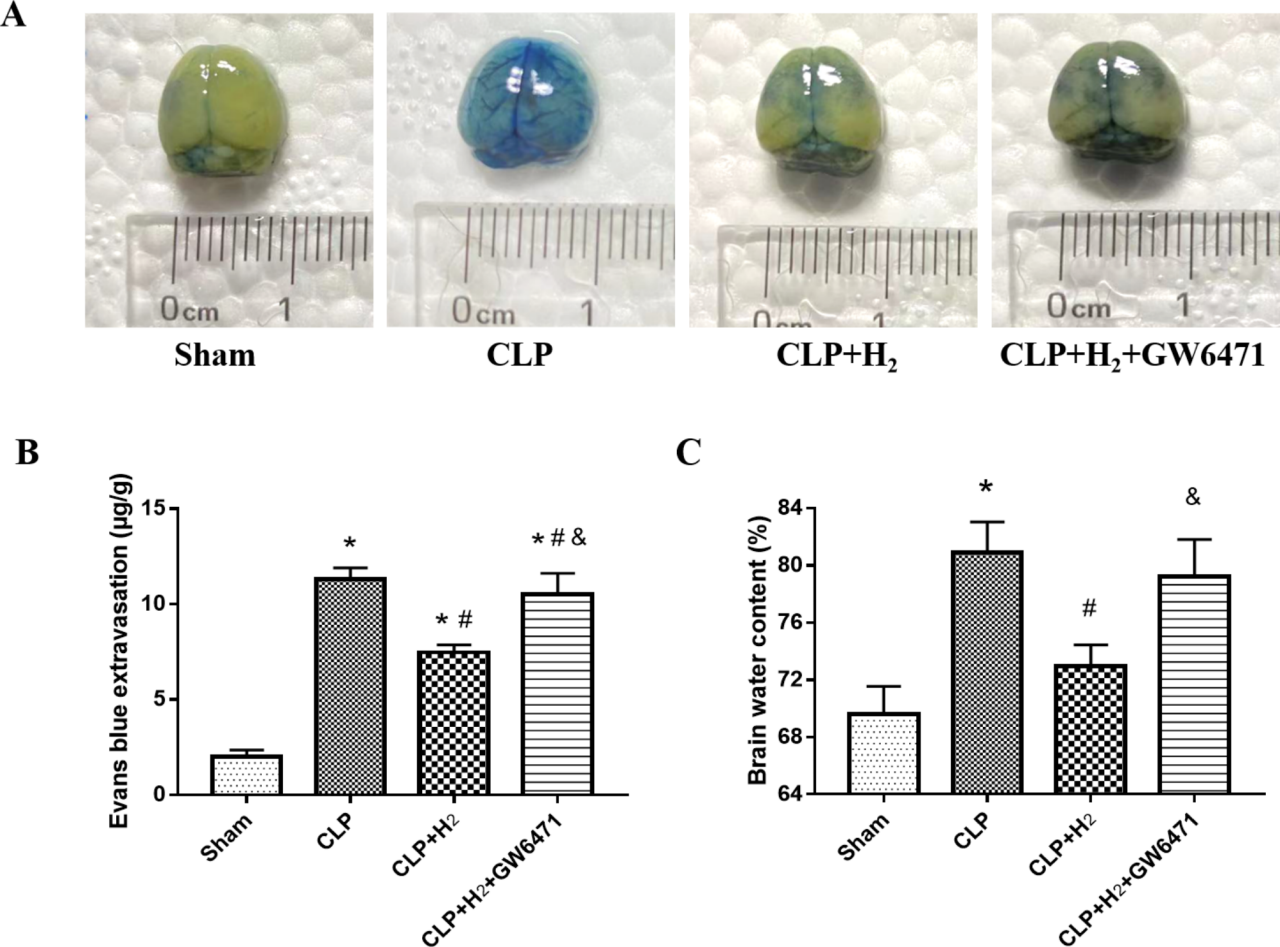
2% H_2_ inhalation correlated with PPARα alleviates the brain oedema and BBB disruption induced by sepsis in mice. **(A)**. Representative images of mouse brains were observed at 24 h after the operation, and Evans blue (EB) leakage (blue colour) is evidence of BBB disruption at 24 h after the CLP operation. **(B)** EB extravasation in brain tissues of each group was quantified; **(C)**. Brain water contents in mice of each group were measured by a dry‒wet method. Values are expressed as the mean ± SD (n = 4 mice per group). *P < 0.05 vs. Sham group; #P < 0.05 vs. CLP group; &P < 0.05 vs. CLP + H_2_ group

Evans blue (EB), which is a nontoxic dye, can bind to serum albumin and rarely cross the BBB. Once the BBB is disrupted, some albumin bound to EB can infiltrate the brain. Thus, EB extravasation could be used to evaluate the severity of BBB disruption. Additionally, brain water content can also be regarded as an indicator of the disruption of the BBB. At 24 h postoperation, marked increases in the quantity of EB were observed in the CLP, CLP + H_2_ and CLP + H_2_ + GW6471 groups compared to the sham group, as shown in Fig. 5A and B (P < 0.001). In the CLP, CLP + H_2_ and CLP + H_2_ + GW6471 groups, the brain water content was also increased compared to that in the sham group (Fig. 5C, P < 0.01). After 2% H_2_ inhalation, the EB extravasation and brain water contents of the CLP + H_2_ group with the upregulation of PPARα were decreased significantly compared to those in the CLP + H_2_ + GW6471 and CLP groups (P < 0.001). The EB extravasation and water content in the brain revealed that the effect of 2% H_2_ lightening the disruption of the BBB in septic mice was related to PPARα expression.

### The effects of hydrogen on BBB impairment in septic mice are mediated by PPARα, which regulates ABC efflux transporters

**Fig. 6:**
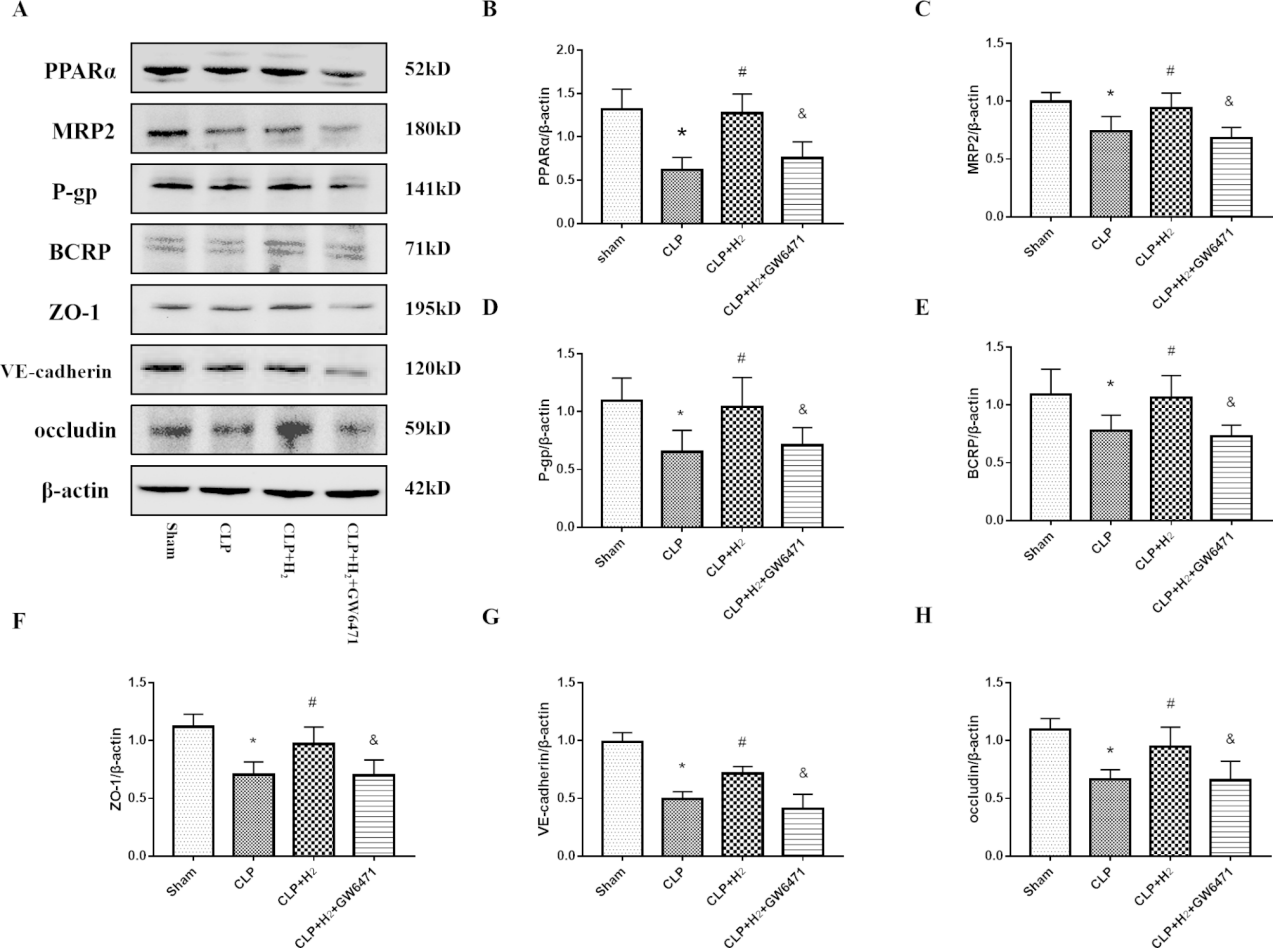
Treatment with 2% H_2_ and PPARα upregulation significantly increased the expression of ABC efflux transporters (P-gp, MRP2, BCRP) and BBB permeability-associated proteins (ZO-1, occludin, VE-cadherin) in CLP-induced septic mice. **(A)** The expression of PPARα, MRP2, P-gp, BCRP, ZO-1, VE-cadherin and occludin in the cortex tissues of septic mice was detected by western blot. Quantitative analysis of **(B)** PPARα, **(C)** MRP2, **(D)** P-gp, **(E)** BCRP, **(F)** ZO-1, **(G)** VE-cadherin and **(H)** occludin are shown as percentage changes of the protein level compared to sham levels. n = 6 mice per group, *P < 0.05 vs. the sham group; #P < 0.05 vs. the CLP group; &P < 0.05 vs. the CLP + H_2_ group

ABC transporters have been regarded as one of a variety of factors related to disorder pathophysiology. ABC transporters are particularly important in barrier tissues, including the BBB, and transport endogenous and exogenous molecules in the CNS to regulate homeostasis [40, 41]. ABC transporter dysfunction is linked to neurological disorders, according to increasing evidence [41, 42]. Additionally, clofibrate (the ligand of PPARα) has been shown to increase the expression and activity of Bcrp in cultured human brain capillary endothelial cells, and BBB efflux transporters may be the targets of PPARα [28]. Furthermore, recent research suggested that PPARα activation could increase P-glycoprotein, Bcrp and Mrp2 activity and expression at the BBB [9]. Therefore, we investigated this topic in sepsis using immunoblotting (Fig. 6A) and densitometry data (Fig. 6B-H) in all groups. Our previous study illustrated that the level of PPARα was reduced in the brain tissue in CLP mice compared with sham mice and upregulated in the CLP group treated with hydrogen [30] (P < 0.05, Fig. 6A-B). As shown in the figures, ABC transporter (P-gp, MRP2 and BCRP) and BBB permeability protein (ZO-1, VE-cadherin, occludin) expression in CLP mice was decreased compared to that in the sham group (P < 0.05, Fig. 6). Compared to the CLP + H_2_ group, the CLP group showed reduced ZO-1, VE-cadherin, and occludin expression, and both of these groups showed higher expression of all three proteins than the CLP + H_2_ + GW6471 group (P < 0.05, Fig. 6A, F-G). We also found increased levels of P-gp, MRP2 and BCRP in the CLP + H_2_ group compared to the CLP group (P < 0.05). The expression of P-gp, MRP2 and BCRP in the CLP + H_2_ + GW6471 group was decreased compared to that in the CLP + H_2_ group (P < 0.05, Fig. 6A, C-E). Taken together, the results showed that increasing the level of PPARα could upregulate the levels of ZO-1, VE-cadherin, occludin and ABC transporters (P-gp, MRP2 and BCRP) in H_2_-treated mice with CLP.

### The increasing level of PPARα improves the level of ABC transporters and BBB permeability proteins in the LPS-treated b. End3 cells were cultured with a hydrogen-rich medium

**Fig. 7:**
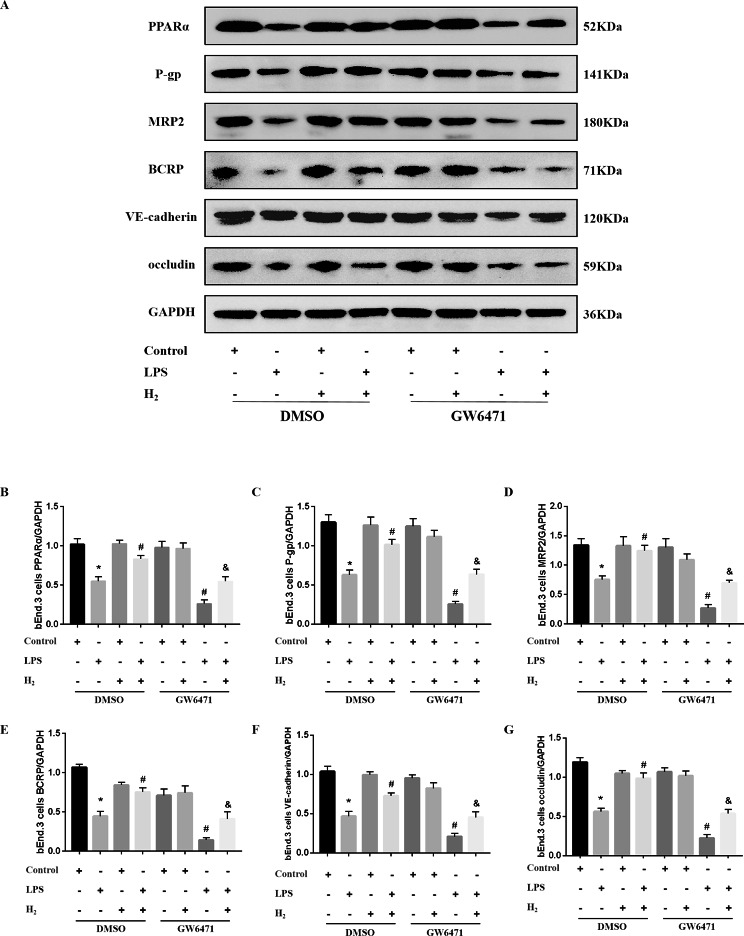
Positive effects of H_2_ on BBB-related protein expression in LPS-treated bEnd.3 cells cultured with GW6471- or DMSO-added medium. **(A)** The expression levels of PPARα, P-gp, MRP2, BCRP, VE-cadherin and occludin were detected by western blotting. Quantitative analysis of **(B)** PPARα/GAPDH, **(C)** P-gp/GAPDH, **(D)** MRP2/GAPDH, **(E)** BCRP/GAPDH, **(F)** VE-cadherin/GAPDH, and **(G)** occludin/GAPDH is represented as the percentage change in the protein level compared to control levels. n = 6 samples per group, *P < 0.05 vs. control group; #P < 0.05 vs. LPS group; &P < 0.05 vs. LPS + H_2_ group

In vitro, PPARα expression decreased markedly in the LPS + DMSO group compared to the control + DMSO group (P < 0.05), and the medium with hydrogen increased the level of PPARα in the LPS + H_2_ + DMSO group compared to the LPS + DMSO group (P < 0.05). Compared to LPS + H_2_ + DMSO treatment alone, LPS + H_2_ + GW6471 treatment markedly reduced the level of PPARα (P < 0.05). Moreover, PPARα expression was lower in the LPS + GW6471 group than in the LPS + DMSO group (P < 0.05) (Fig. 7A-B). P-gp, MRP2 and BCRP were decreased in the LPS + DMSO group compared with the control + DMSO group (P < 0.05) and were upregulated in the LPS + H_2_ + DMSO group compared to the LPS + DMSO group (P < 0.05) (Fig. 7A-E). In addition, the P-gp, MRP2 and BCRP levels were lower in the LPS + GW6471 group than in the LPS + DMSO group (P < 0.05). Compared to the LPS + H_2_ + DMSO group, P-gp, MRP2 and BCRP were reduced in the LPS + H_2_ + GW6471 group (P < 0.05) (Fig. 7A-E). Compared to the control + DMSO group, LPS treatment decreased the expression of VE-cadherin and occludin in the LPS + DMSO group (P < 0.05). Additionally, medium with hydrogen increased VE-cadherin and occludin expression in the LPS + H_2_ + DMSO group compared to the LPS + DMSO group (P < 0.05). There was less VE-cadherin and occludin in the LPS + GW6471 group than in the LPS + DMSO group (P < 0.05). Furthermore, GW6471 treatment in the LPS + H_2_ + GW6471 group mediated a decrease in VE-cadherin and occludin compared to the LPS + H_2_ + DMSO group (P < 0.05) (Fig. 7A F, 7G).

## Discussion

As a syndrome of severe systemic inflammation, sepsis is due to a variety of infectious elements [43] and results in an impaired BBB, which leads to brain injury, including cognitive impairment and behavioural defects, called SAE [4, 44]. Our previous research found that H_2_ could mainly prevent multiple organ dysfunction syndrome (MODS), including brain damage, rather than sepsis [21, 22]. Increasing evidence has demonstrated that the integrity of the BBB is destroyed once sepsis occurs. Recently, research has indicated that BBB permeability increases within 24 h in the cortex tissues, hippocampus, thalamus and perirhinal cortex of rats with lipopolysaccharide (LPS) (10 mg/kg in 100 µL saline) intraperitoneal (i.p.) injection compared to saline-treated animals [45]. In addition, human brain tissues of patients with decreased sepsis showed that the TJ proteins occludin, claudin-5, VE-cadherin and zonula occludens-1 (ZO-1) in microvascular endothelial cells were downregulated [46], illustrating BBB dysfunction. Because the damage to the BBB was due mainly to sepsis and SAE pathophysiology, the CNS defence becomes more vulnerable to neurotoxic factors, including free radicals, intravascular proteins, inflammatory cytokines, plasma, circulating leukocytes and so on [14, 15]. BBB damage in SAE involves several mechanisms, so the therapeutic factors are also more complex. Previously, we demonstrated that H_2_ treatment protects the BBB by reducing increased permeability, alleviating SAE and alleviating cognitive impairment, which is induced by Nrf2 and its signalling pathways [32]. We also demonstrated that H_2_ treatment provides vital protection against SAE-induced brain damage, which is closely related to PPARα in the CREB-BDNF pathway and the level of its related molecules [30]. Strikingly, the results revealed that the activation of PPARα could upregulate transporter activity and the expression of ABC transporters, which are highly expressed in the BBB and transport many exogenous and endogenous substrates from inside the cell to maintain homeostasis in the central nervous system [47–49]. While there is evidence that pioglitazone (an agonist of the PPARγ pathway) attenuates the effects of peripheral inflammation in a human in vitro BBB model [50], there are no studies about PPARα on the BBB related to hydrogen. Therefore, we explored the PPARα regulatory effects on ABC transporters, which were upregulated by molecular hydrogen, on LPS- and CLP-mediated BBB disruption in the two experimental models.

In vitro experiments using bEnd.3 cells (microvascular endothelial cells) revealed that the expression of PPARα, VE-cadherin and occludin was downregulated with LPS treatment in FBS-free DMEM supplemented with GW6471 (a PPARα inhibitor) or DMSO but increased in the LPS + H2 + DMSO group and decreased in the LPS + H2 + GW6471 group. As a constituent of the BBB, ABC transporters (P-gp, MRP2 and BCRP) were reduced in the LPS + DMSO group compared to the control group and increased in the LPS + H2 + DMSO group compared to the LPS + DMSO group. GW6471 (an inhibitor of PPARα) treatment in LPS + H_2_ cells decreased the P-gp, MRP2 and BCRP levels. The results showed that VE-cadherin, occludin and ABC transporters (P-gp, MRP2 and BCRP) were regulated by PPARα in bEnd.3 cells treated with LPS in hydrogen-rich FBS-free DMEM.

As in previous studies, we selected the CLP model for this study because it is recognized widely as the gold standard model of sepsis [51]. To assess the positive effects of H_2_ with PPARα in CLP mice, we performed inhalation of 2% H_2_ for 60 min at 1 h and 6 h post-surgery in the CLP + H_2_ + GW6471 and CLP + H_2_ groups with GW6471 injection or DMSO at 1 h preoperatively and recorded the survival rates. 2% H_2_ improved the survival rates in the CLP + H_2_ group compared with the CLP + H_2_ + GW6471 and CLP groups. Furthermore, after H_2_ treatment, the changes and concentration of H_2_ were measured in the brains of mice. The data suggested that the concentration of H_2_ increased with the duration of inhalation and peaked point at 45 min, proving that inhaled H_2_ can pass through the BBB into brain tissues. The increasing release of inflammatory mediators, including IL-6, TNF-α, IL-1β and HMGB1, one of the important factors causing brain damage [52], was tested to assess the hydrogen effect on SAE. As a result, H_2_ decreased the levels of IL-1β, TNF-α, IL-6 and HMGB1 in the cortex of the CLP + H_2_ group compared with the CLP and CLP + H_2_ + GW6471 groups. We also observed that the increasing level of PPARα induced by hydrogen could ameliorate the neurobehavioural abnormalities (Y-maze, fear conditioning test) of septic mice in the CLP + H_2_ group compared to the CLP and CLP + H_2_ + GW6471 groups. Nissl staining, TUNEL staining and immunofluorescence staining were used to observe and compare brain injury in our study. In addition, we measured the extravasation of Evans blue and water content in the brain to assess the effects of BBB damage in sepsis. Then, we found that H_2_ treatment can reduce the abovementioned indicators to relieve BBB damage, which was related to PPARα upregulation. BBB permeability is associated with cell junction protein changes in endothelial cells [44]. Western blot analysis illustrated that the levels of tight junction proteins (ZO-1 and occludin) in the cortex of the CLP + H_2_ group were increased compared to those in the CLP and CLP + H_2_ + GW6471 groups. We also found that the ABC transporters (P-gp, MRP2 and BCRP) that constitute the BBB were mediated by PPARα in 2% H_2_-injured septic mice and decreased the permeability of the BBB to alleviate brain damage in SAE. Importantly, our data demonstrated that the protective effects of hydrogen on the BBB were mediated through PPARα-mediated regulation of ABC transporters.

In summary, our data demonstrated that LPS/CLP-induced brain damage was associated with dysfunction of tight junction proteins and ABC transporters, leading to increased permeability and destruction of the BBB and resulting from cognitive impairment; nevertheless, hydrogen reversed the damage in septic mice via increased expression of PPARα, which regulates ABC transporters. However, although our studies provide important information about hydrogen therapy in sepsis, our experiments have only been performed in mice or using mouse cells. Thus, more studies are needed to elucidate the therapeutic mechanistic details of H_2_ in sepsis patients, and it is still an urgent problem before H_2_ can be applied in clinical treatment.

## Conclusion

Taken together, our results provide compelling evidence that inhaling 2% H_2_ could have a vital effect in the protection of BBB permeability and neuroinflammation, thereby improving the brain damage of sepsis and alleviating cognitive dysfunction, which may be mediated by PPARα regulating ABC transporters. Thus, we speculate that H_2_ treatment would be a promising therapy for alleviating SAE in the future.

## Electronic supplementary material

Below is the link to the electronic supplementary material.

Supplementary Material 1

## Data Availability

The data used to support the findings of this study are available from the corresponding author upon request.

## Declarations

### ARRIVE statement

The study is reported in accordance with ARRIVE guidelines (https://arriveguidelines.org).
