# Supplementary material for: Hydrogen alleviated cognitive impairment and blood‒brain barrier damage in sepsis-associated encephalopathy by regulating ABC efflux transporters in a PPARα-dependent manner
Source: BMC Neurosci. 2023 Jul 20;24:37. doi: 10.1186/s12868-023-00795-3 (PMC10360271; doi:10.1186/s12868-023-00795-3)
Supplement: Supplementary file 1 — Supplementary Material 1 [file 12868_2023_795_MOESM1_ESM.docx]

**Hydrogen alleviated cognitive impairment and blood‒brain barrier damage in sepsis-associated encephalopathy by regulating ABC efflux transporters in a PPARα-dependent manner**

**List of Authors:**

Yuanyuan Bai^a,^**^*^** (Baiyuanyuan0523@tmu.edu.cn)

Wen Mi^b,^**^*^** (598515914@qq.com)

Xiaoyin Meng^c,^**^*^** (savagemeng@qq.com)

Beibei Dong^a^ (senyu1219@163.com)

Yi Jiang^a^ (jiangyi@tmu.edu.com)

Yuechun Lu^d,^**^#^** [(luyuechun@sina.com](mailto:(daguang521521@163.com))

Yonghao Yu^a,^**^#^** [(yyu@tmu.edu.cn)](mailto:(yyu@tmu.edu.cn))

^a^Department of Anesthesiology, Tianjin Medical University General Hospital, Tianjin, China, 300052

^b^Department of Anesthesiology, Tianjin Baodi Hospital, Baodi Clinical College of Tianjin Medical University, Tianjin 301800, China.

^c^Department of Gynecology and Obstetrics, Tianjin Medical University General Hospital, Tianjin, China, 300052

^d^Department of Anesthesiology, The Second Hospital of Tianjin Medical University, Tianjin, China, 300211

**^*^**Yuanyuan Bai , Wen Mi and Xiaoyin Meng contributed equally to this work. **^#^**Yuechun Lu and Yonghao Yu were the co-corresponding authors.

**^#^Address correspondence to:**

**Yonghao Yu, MD, PhD**

Department of Anesthesiology, Tianjin Institute of Anesthesiology, General Hospital of Tianjin Medical University, No. 154 Anshan Road, Heping District, Tianjin 300052, PR China.

Telephone: +86 22-60361510

Fax: +86 22-27813550

E-mail: yyu@tmu.edu.cn

**Supplemental Materials**

**Figure S1：**The original WB images of PPARα in the Sham, CLP, CLP+H_2_ and CLP+H_2_+GW6471 groups in vivo were shown in figure A-F.

**
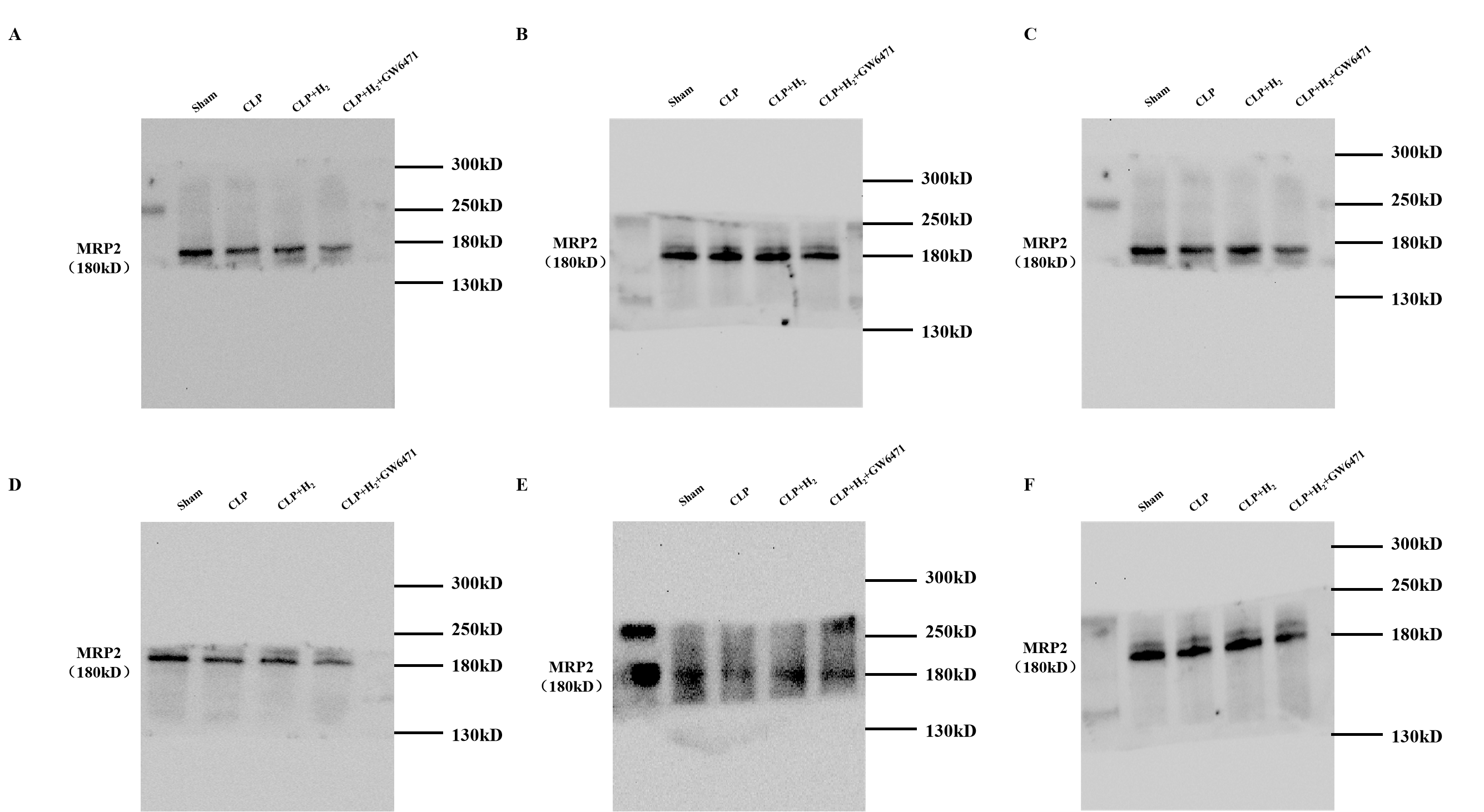
**

**Figure S2：**The original WB images of MRP2 in the Sham, CLP, CLP+H_2_ and CLP+H_2_+GW6471 groups in vivo were shown in figure A-F.


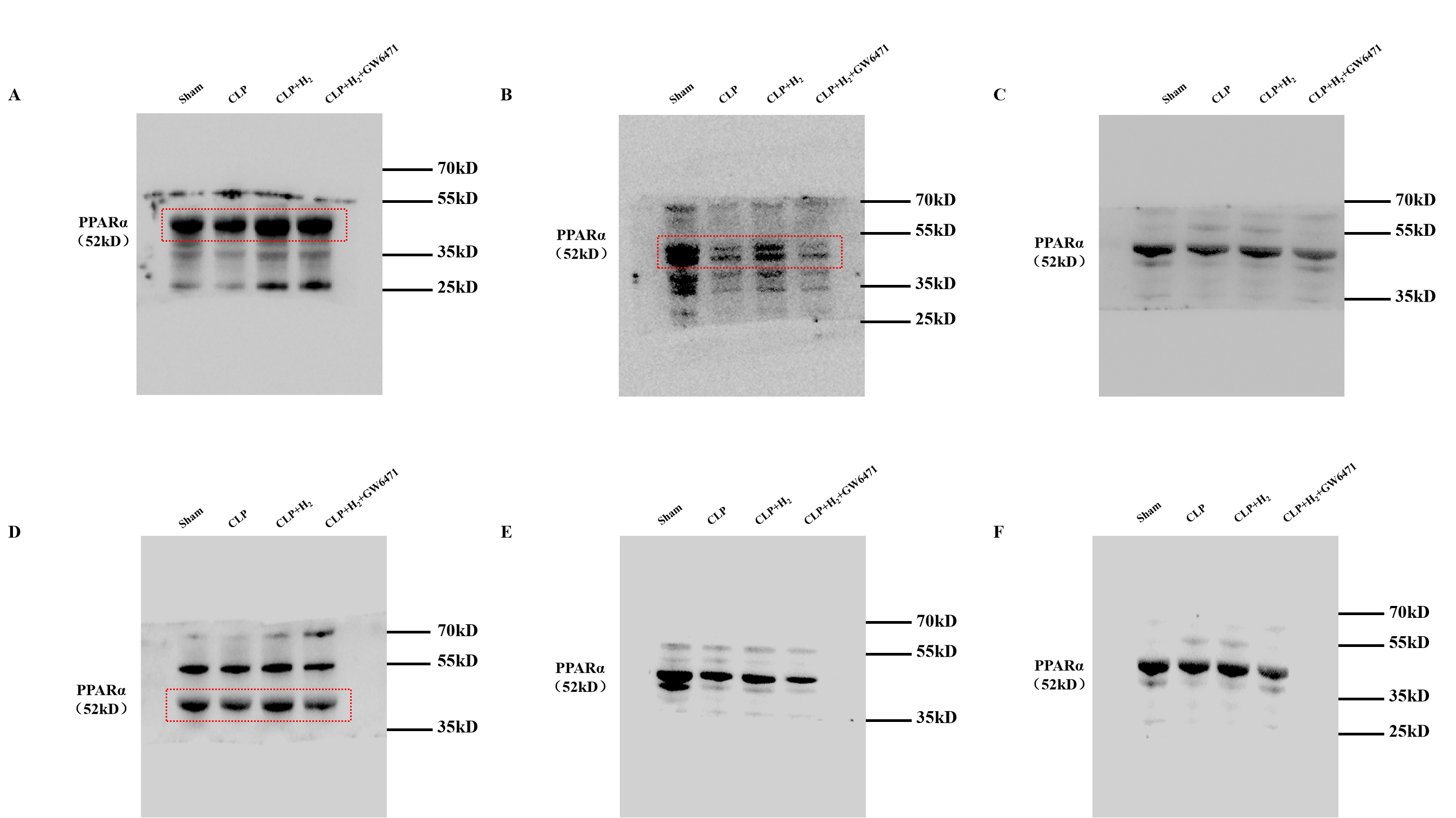


**
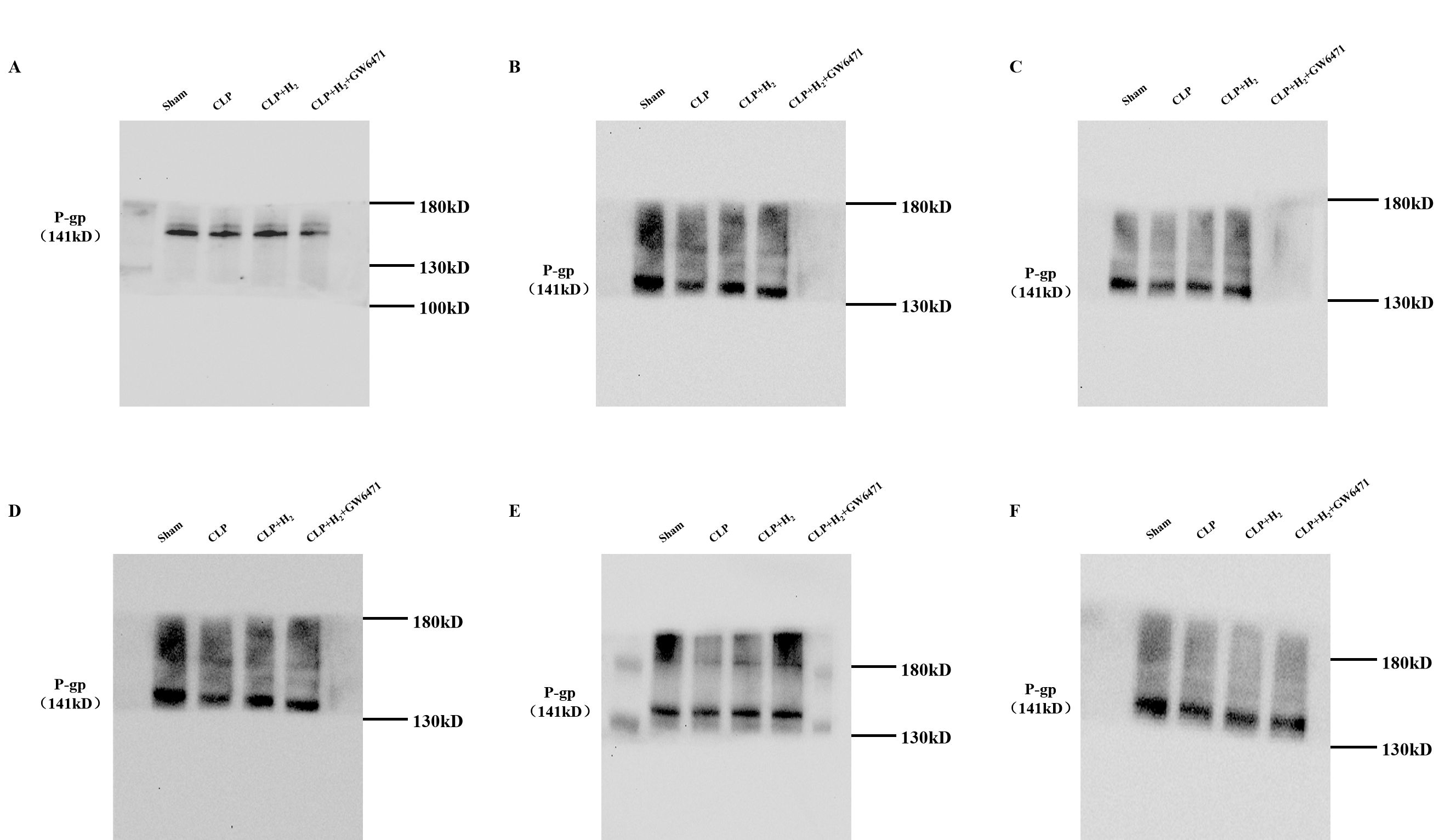
**

**Figure S3：**The original WB images of P-gp in the Sham, CLP, CLP+H_2_ and CLP+H_2_+GW6471 groups in vivo were shown in figure A-F.

**
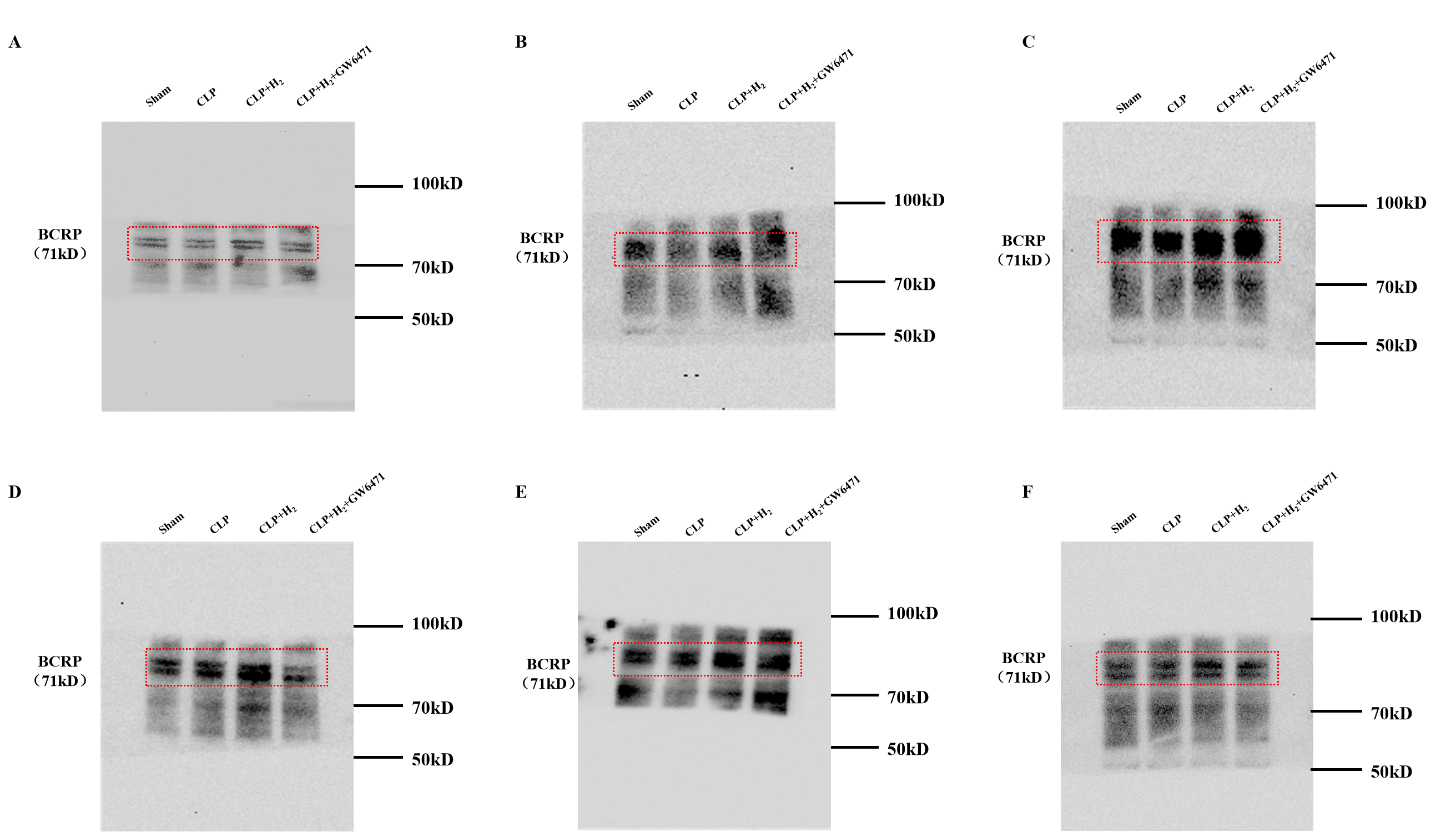
**

**Figure S4：**The original WB images of BCRP in the Sham, CLP, CLP+H_2_ and CLP+H_2_+GW6471 groups in vivo were shown in figure A-F.

**
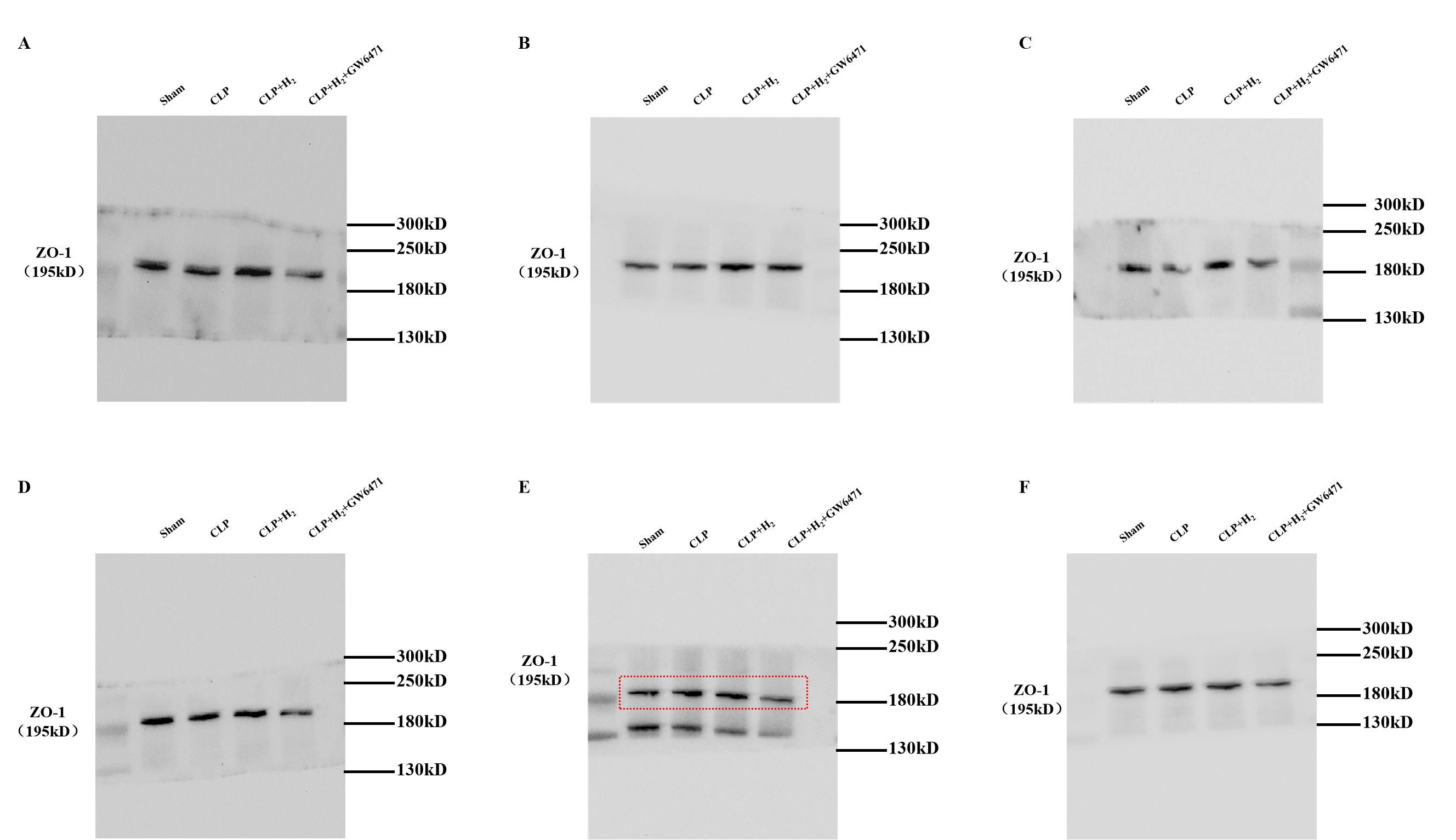
**

**Figure S5：**The original WB images of ZO-1 in the Sham, CLP, CLP+H_2_ and CLP+H_2_+GW6471 groups in vivo were shown in figure A-F.

**
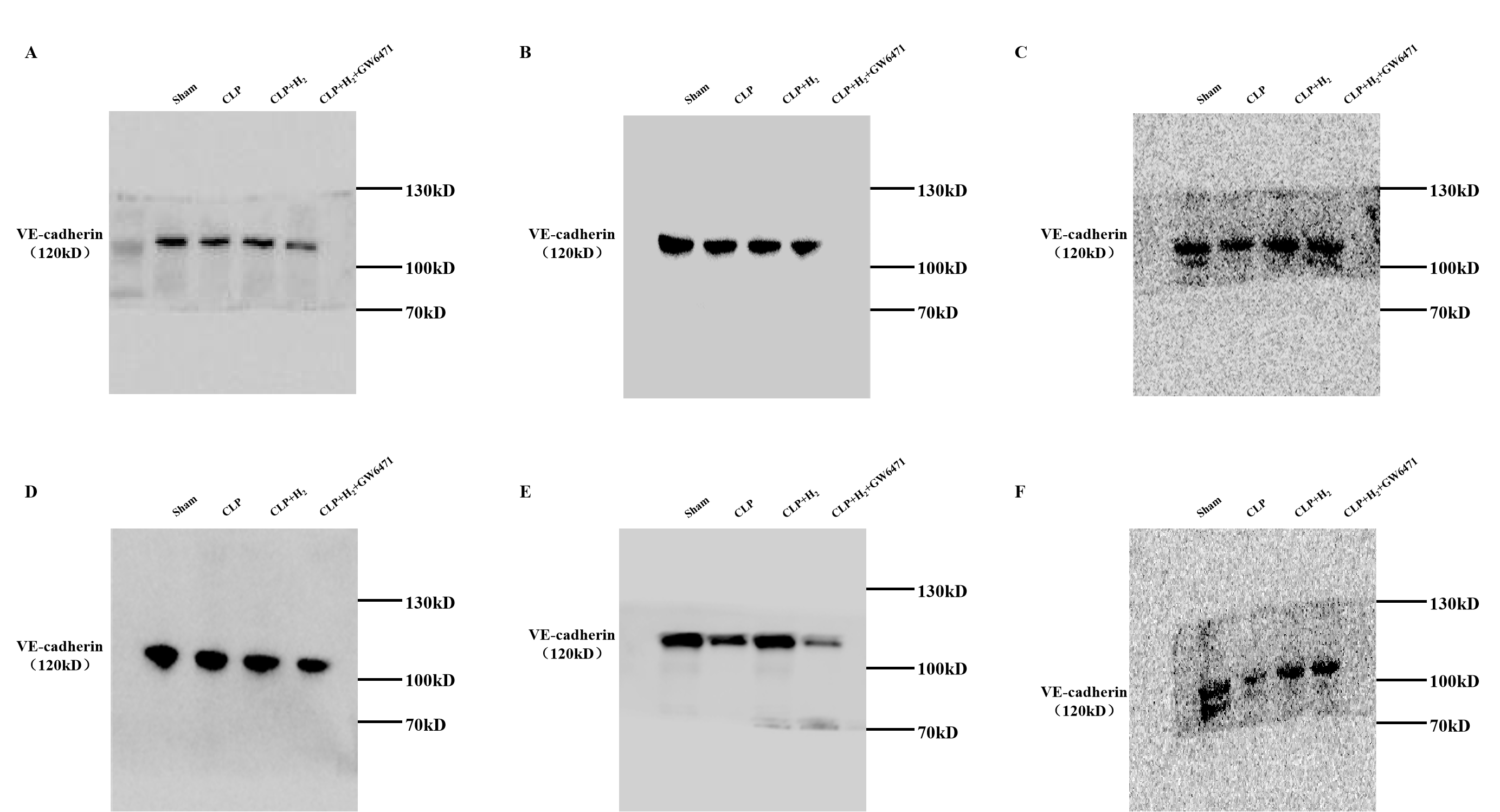
**

**Figure S6：**The original WB images of VE-cadherin in the Sham, CLP, CLP+H_2_ and CLP+H_2_+GW6471 groups in vivo were shown in figure A-F.

**
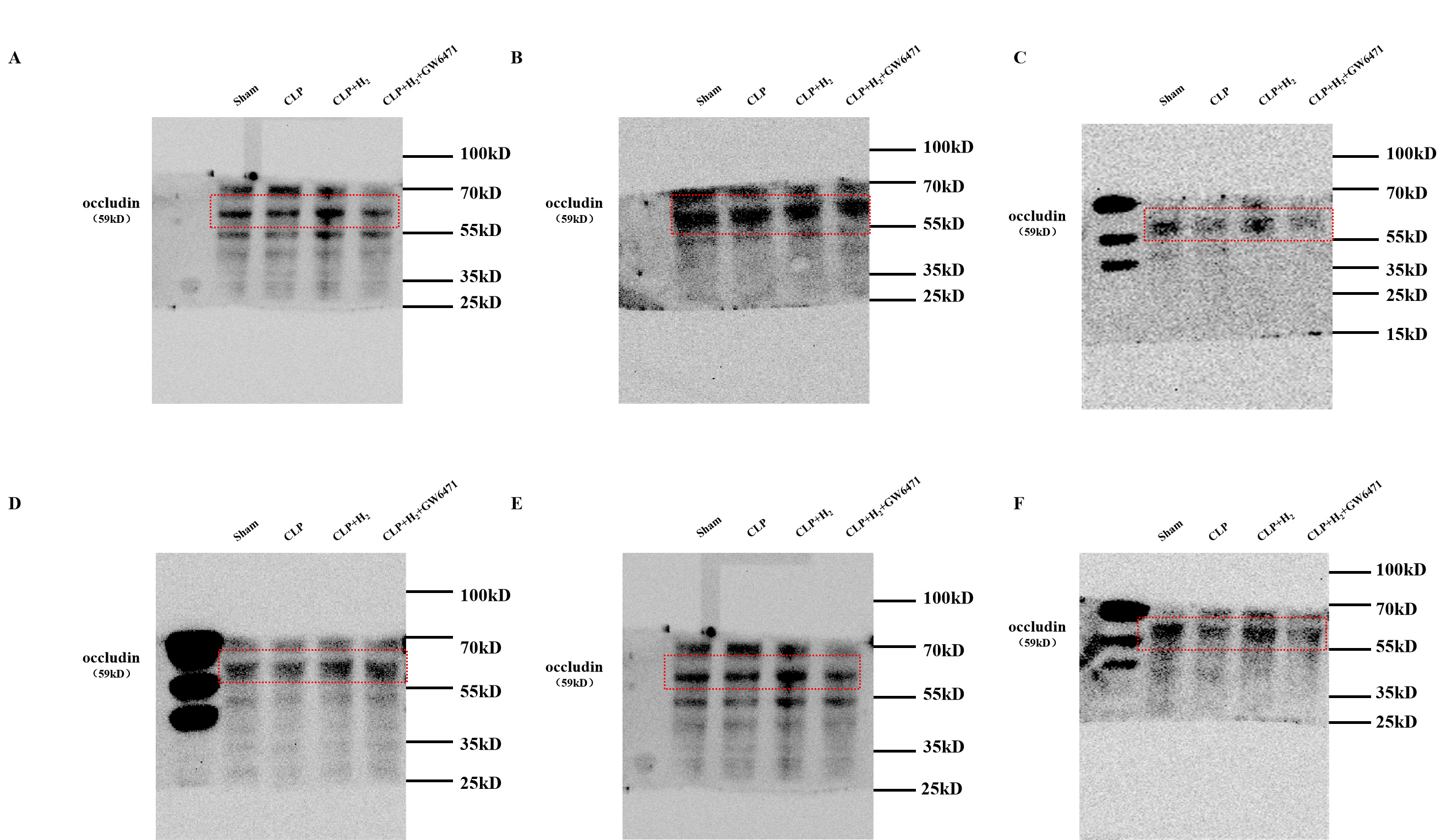
**

**Figure S7：**The original WB images of occludin in the Sham, CLP, CLP+H_2_ and CLP+H_2_+GW6471 groups in vivo were shown in figure A-F.

**
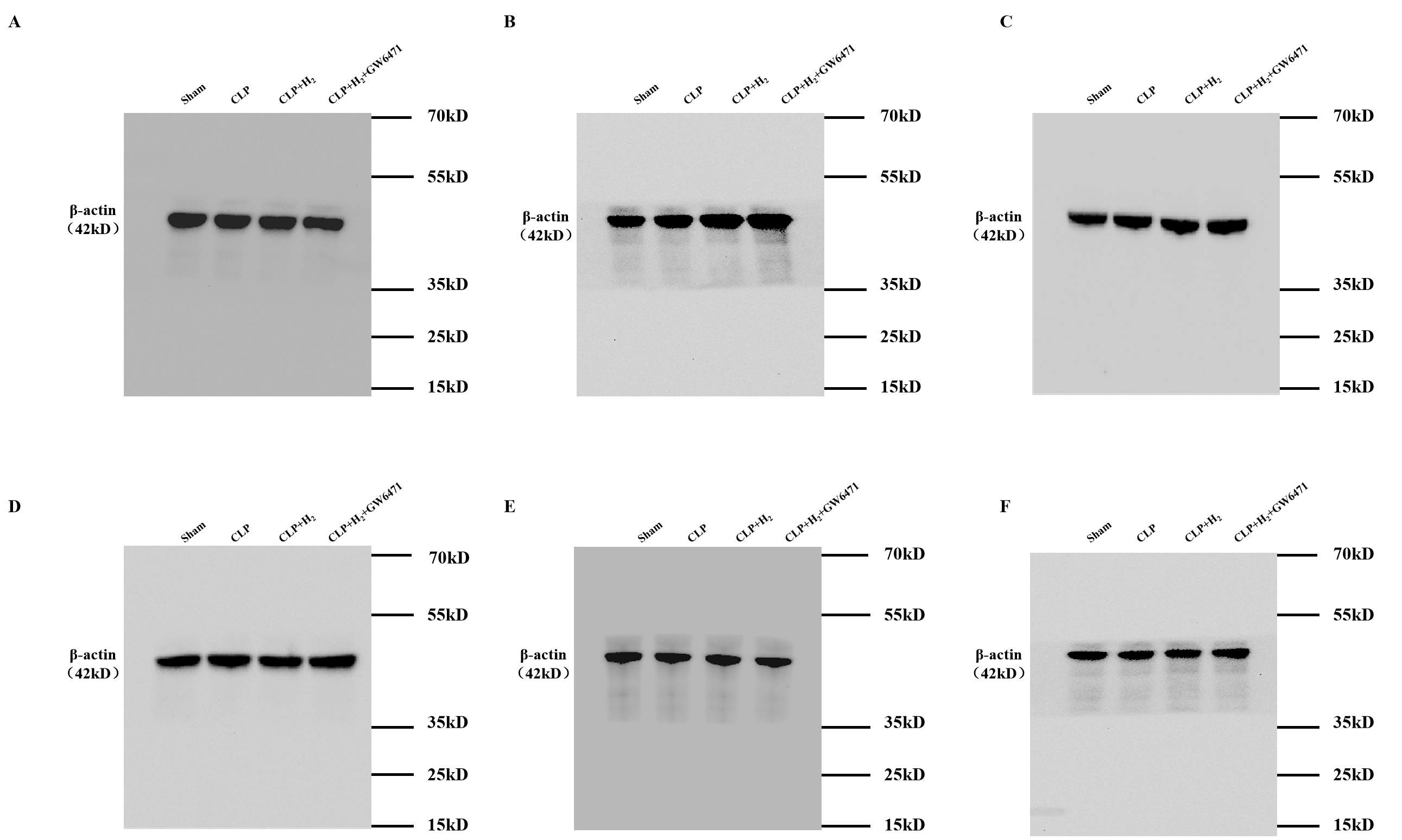
**

**Figure S8：**The original WB images of β-actin in the Sham, CLP, CLP+H2 and CLP+H2+GW6471 groups in vivo were shown in figure A-F.

**
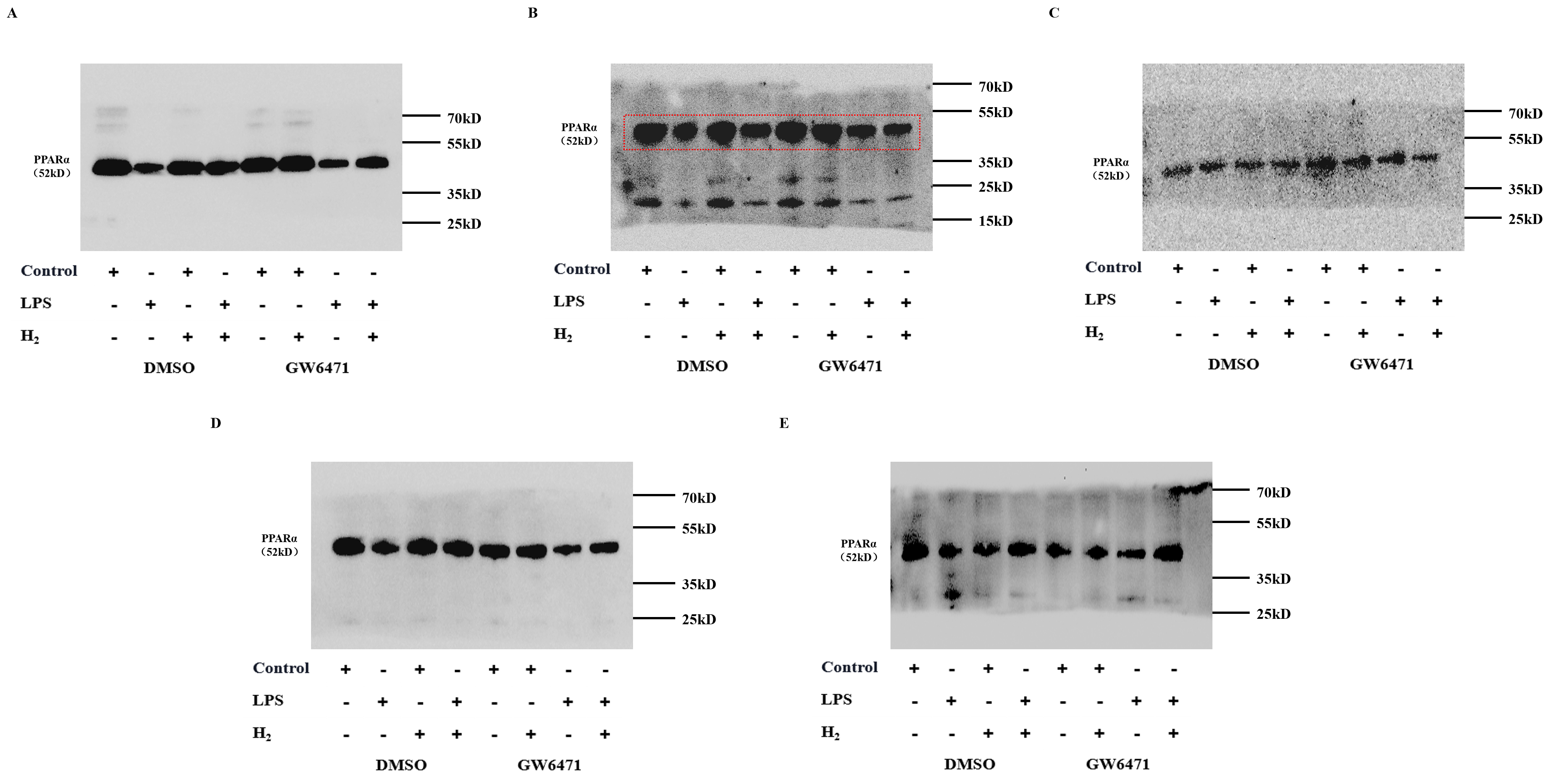
**

**Figure S9：**The original WB images of PPARα in the Control + DMSO, LPS + DMSO, Control + H_2_ + DMSO, LPS + H_2_ + DMSO, Control + GW6471 (PPARα antagonist), LPS + GW6471, Control + H_2_ + GW6471 and LPS + H_2_ + GW6471 groups in vitro were shown in figure A-E.

**
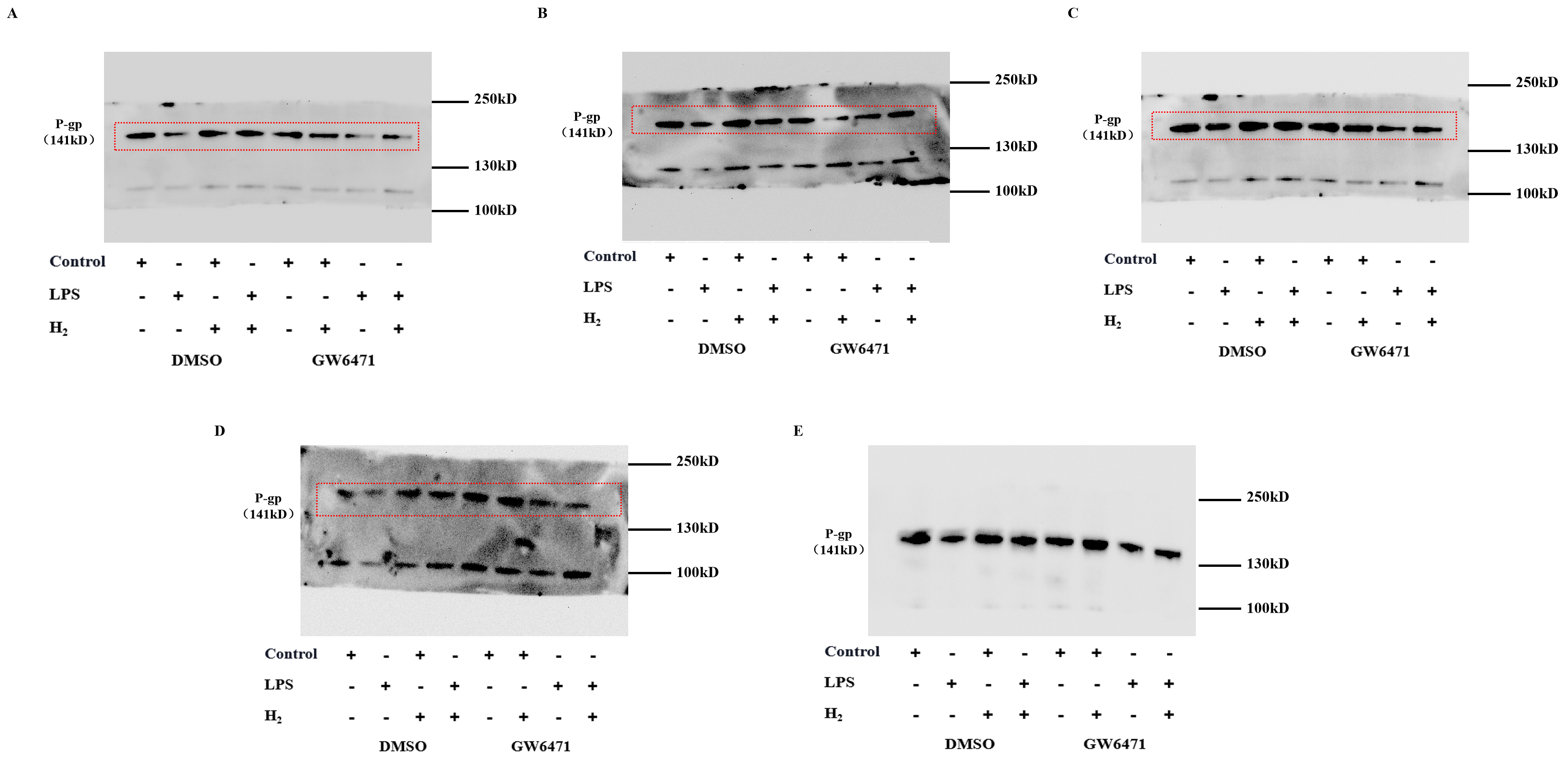
**

**Figure S10：**The original WB images of P-gp in the Control + DMSO, LPS + DMSO, Control + H_2_ + DMSO, LPS + H_2_ + DMSO, Control + GW6471 (PPARα antagonist), LPS + GW6471, Control + H_2_ + GW6471 and LPS + H_2_ + GW6471 groups in vitro were shown in figure A-E.

**
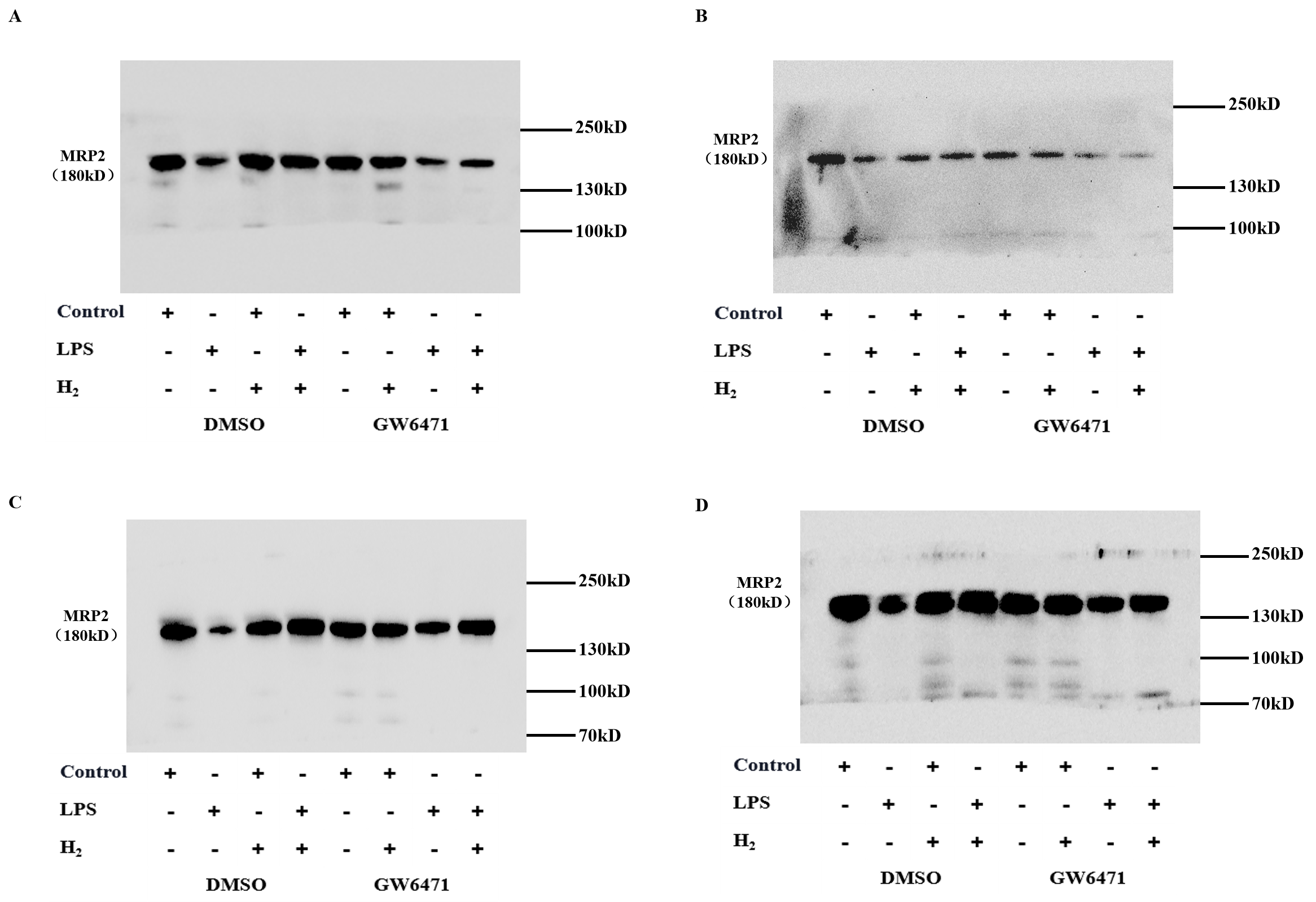
**

**Figure S11：**The original WB images of MRP2 in the Control + DMSO, LPS + DMSO, Control + H_2_ + DMSO, LPS + H_2_ + DMSO, Control + GW6471 (PPARα antagonist), LPS + GW6471, Control + H_2_ + GW6471 and LPS + H_2_ + GW6471 groups in vitro were shown in figure A-D.

**
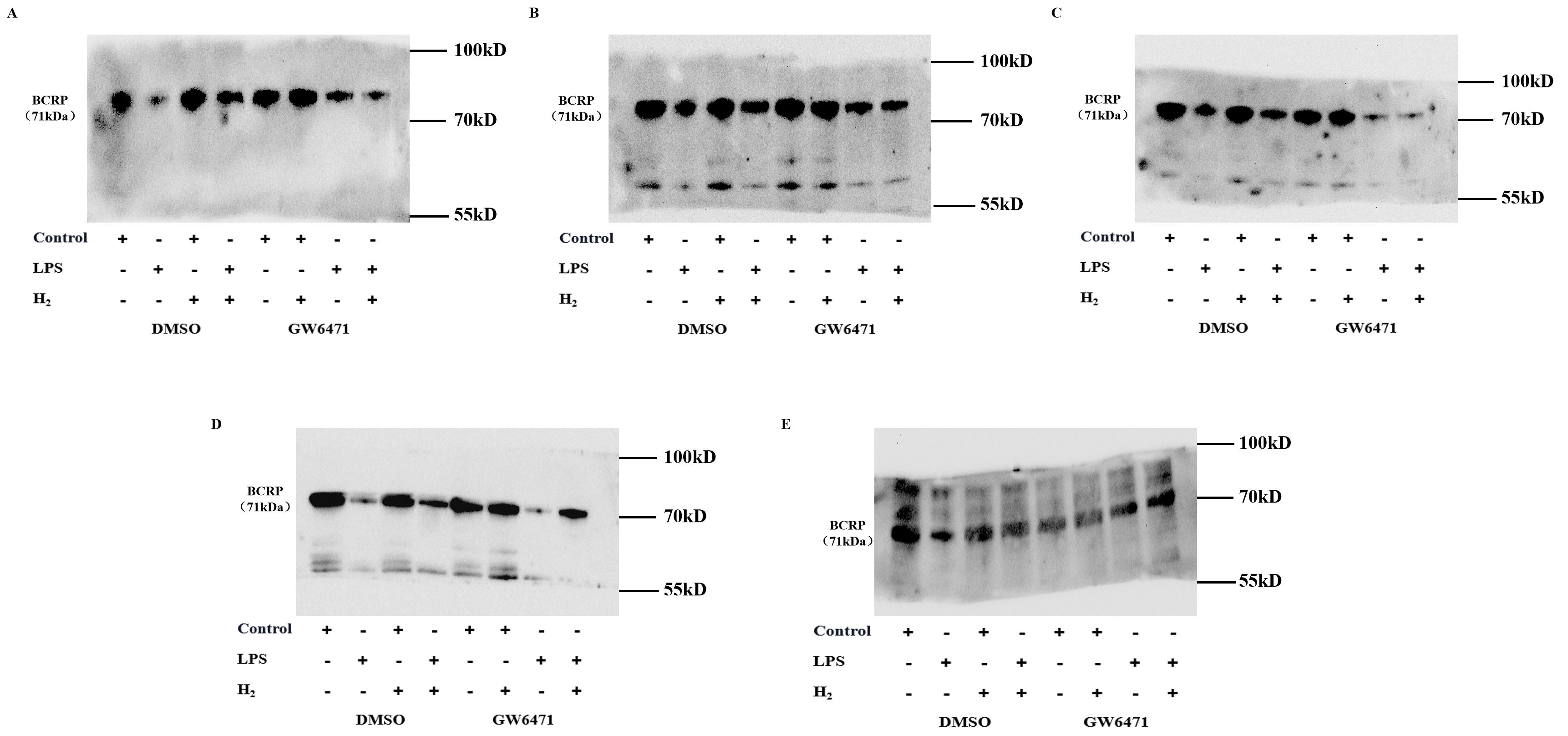
**

**Figure S12：**The original WB images of BCRP in the Control + DMSO, LPS + DMSO, Control + H_2_ + DMSO, LPS + H_2_ + DMSO, Control + GW6471 (PPARα antagonist), LPS + GW6471, Control + H_2_ + GW6471 and LPS + H_2_ + GW6471 groups in vitro were shown in figure A-E.

**
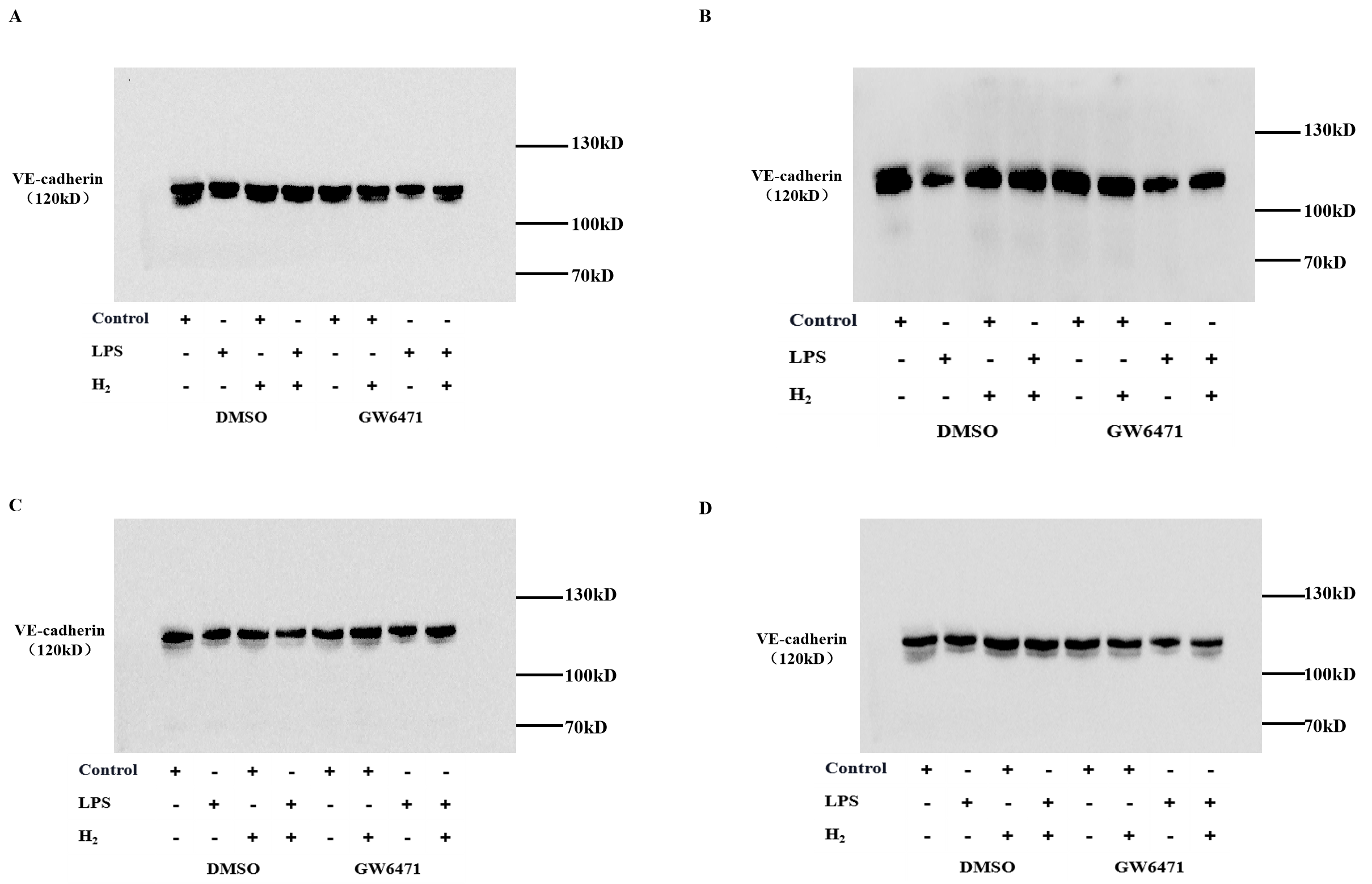
**

**Figure S13：**The original WB images of VE-cadherin in the Control + DMSO, LPS + DMSO, Control + H_2_ + DMSO, LPS + H_2_ + DMSO, Control + GW6471 (PPARα antagonist), LPS + GW6471, Control + H_2_ + GW6471 and LPS + H_2_ + GW6471 groups in vitro were shown in figure A-D.

**
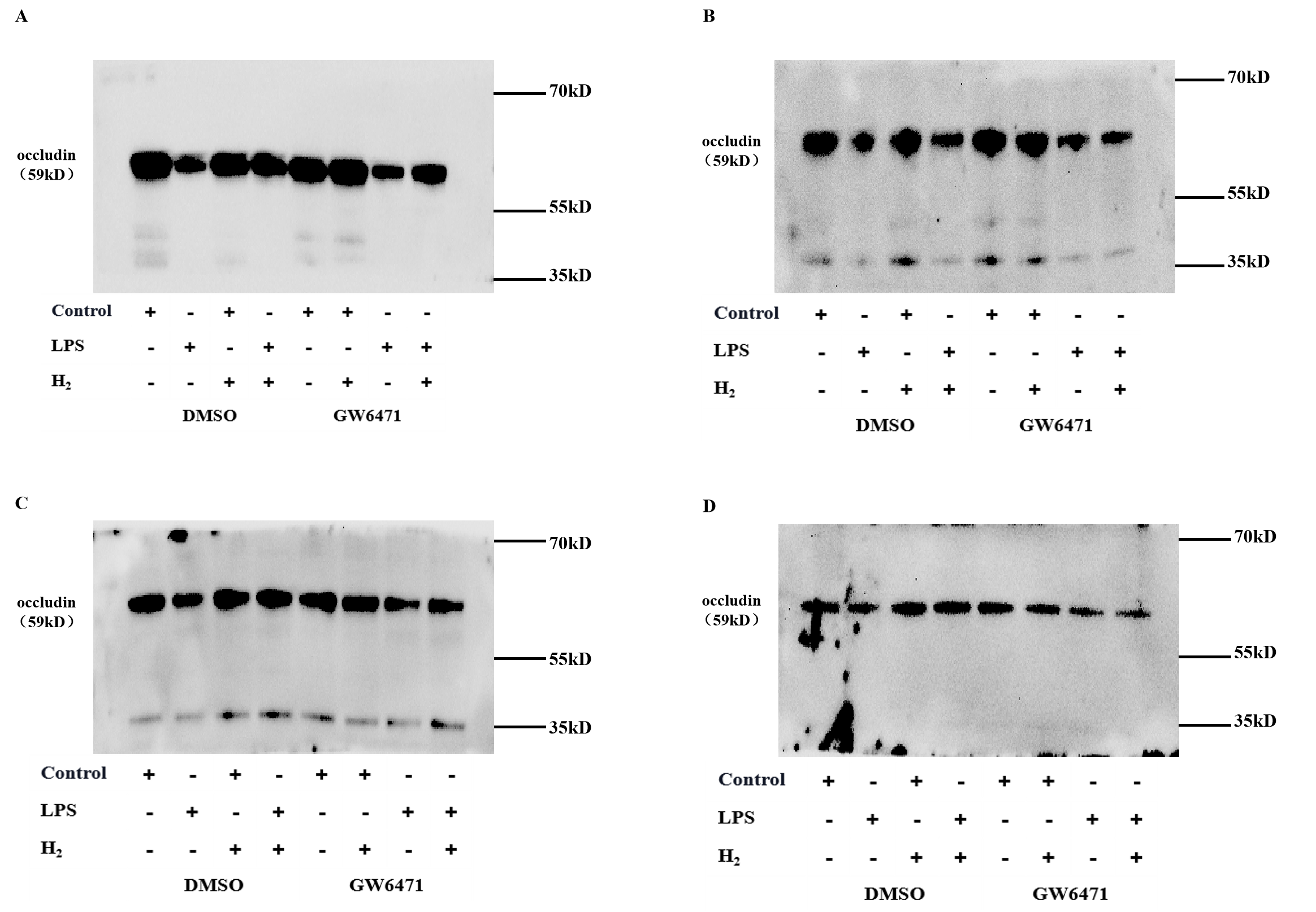
**

**Figure S14：**The original WB images of occludin in the Control + DMSO, LPS + DMSO, Control + H_2_ + DMSO, LPS + H_2_ + DMSO, Control + GW6471 (PPARα antagonist), LPS + GW6471, Control + H_2_ + GW6471 and LPS + H_2_ + GW6471 groups in vitro were shown in figure A-D.

**
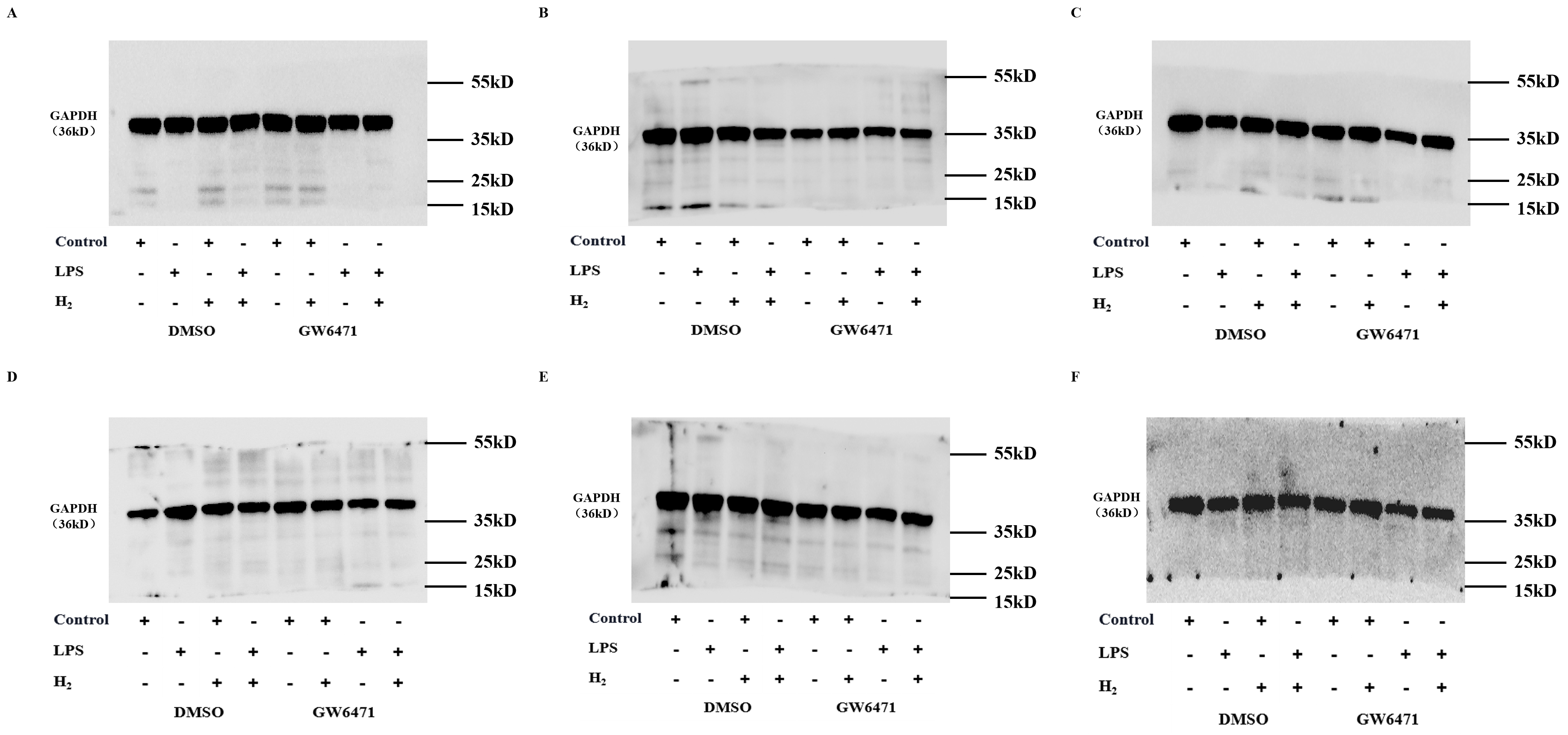
**

**Figure S15：**The original WB images of GAPDH in the Control + DMSO, LPS + DMSO, Control + H_2_ + DMSO, LPS + H_2_ + DMSO, Control + GW6471 (PPARα antagonist), LPS + GW6471, Control + H_2_ + GW6471 and LPS + H_2_ + GW6471 groups in vitro were shown in figure A-F.
